# Transcriptional and Post-Transcriptional Regulation of *SPAST*, the Gene Most Frequently Mutated in Hereditary Spastic Paraplegia

**DOI:** 10.1371/journal.pone.0036505

**Published:** 2012-05-04

**Authors:** Brian J. Henson, Wan Zhu, Kelsey Hardaway, Jaime L. Wetzel, Mihaela Stefan, Kathryn M. Albers, Robert D. Nicholls

**Affiliations:** 1 Birth Defects Laboratories, Division of Medical Genetics, Department of Pediatrics, Children’s Hospital of Pittsburgh of UPMC, Pittsburgh, Pennsylvania, United States of America; 2 Department of Human Genetics, University of Pittsburgh Graduate School of Public Health, Pittsburgh, Pennsylvania, United States of America; 3 Department of Medicine, University of Pittsburgh School of Medicine, Pittsburgh, Pennsylvania, United States of America; National Institutes of Health, United States of America

## Abstract

Hereditary spastic paraplegias (HSPs) comprise a group of neurodegenerative disorders that are characterized by progressive spasticity of the lower extremities, due to axonal degeneration in the corticospinal motor tracts. HSPs are genetically heterogeneous and show autosomal dominant inheritance in ∼70–80% of cases, with additional cases being recessive or X-linked. The most common type of HSP is SPG4 with mutations in the *SPAST* gene, encoding spastin, which occurs in 40% of dominantly inherited cases and in ∼10% of sporadic cases. Both loss-of-function and dominant-negative mutation mechanisms have been described for SPG4, suggesting that precise or stoichiometric levels of spastin are necessary for biological function. Therefore, we hypothesized that regulatory mechanisms controlling expression of *SPAST* are important determinants of spastin biology, and if altered, could contribute to the development and progression of the disease. To examine the transcriptional and post-transcriptional regulation of *SPAST*, we used molecular phylogenetic methods to identify conserved sequences for putative transcription factor binding sites and miRNA targeting motifs in the *SPAST* promoter and 3′-UTR, respectively. By a variety of molecular methods, we demonstrate that *SPAST* transcription is positively regulated by NRF1 and SOX11. Furthermore, we show that miR-96 and miR-182 negatively regulate *SPAST* by effects on mRNA stability and protein level. These transcriptional and miRNA regulatory mechanisms provide new functional targets for mutation screening and therapeutic targeting in HSP.

## Introduction

Hereditary spastic paraplegias (HSPs) are a group of genetically heterogeneous neurodegenerative disorders that are characterized by progressive symmetric spasticity of lower extremities [1]–[5]. Neuropathological studies have shown that HSP patients have axonal degeneration of the corticospinal or pyramidal motor and sensory tracts that control the lower extremities [3]–[4], [6]. HSP may be accompanied by muscle weakness, increased stiffness, hyperreflexia, extensor plantar responses, bladder disturbances, and vibratory sense impairment [2], [4]. HSPs are clinically classified as ‘pure’ when they occur with the above features in isolation and ‘complicated’ when they are associated with additional neurological disorders such as mental retardation, amyotrophy, epilepsy, ataxia, deafness, or optic neuropathy [2], [4].

HSPs are genetically heterogeneous with many genes involved in their etiology. Dominant inheritance accounts for ∼70% of HSP, although the mode of inheritance can also be autosomal recessive, X-linked, or sporadic with no familial pattern [2]–[3], [7]. Classification of HSPs is based on their specific chromosomal gene/locus as “SPG” (spastic gait) followed by a progressive number. To date, 48 chromosomal loci have been linked to pathogenesis, although for about half of these the etiological gene remains unidentified [2]–[3], [5], [7]. The most common form of HSP, SPG4, results from various types of mutations in *SPAST*, which occur in 40% of the dominantly inherited cases [3]–[4], [7]–[8]. These mutations include nonsense, missense, and splice mutations that affect the coding sequence (http://www.hgmd.cf.ac.uk/ac/gene.php?gene=SPAST) [9]–[13] as well as deletions or duplications of coding exons [8], [10]–[12], [14]. Mutations in other genes also result in autosomal dominant HSP, including *REEP1* (SPG31), *ATL1* (SPG3A), *KIF5A* (SPG10), *HSPD1* (SPG13), *NIPA1* (SPG6), *KIAA1096* (SPG8), *BSCL2* (SPG17), and *SLC33A1* (SPG42), but the frequency of such occurrences is minor in comparison to *SPAST* mutations [10], [13], [15]–[21].

*SPAST* encodes spastin, which is a member of the AAA (ATPases associated with a variety of cellular activities) protein family [5], [22]–[23]. Spastin possesses microtubule-severing ability, and contributes to membrane modeling, intracellular and axonal transport of vesicles [1], [3], [5], [7]. It has been suggested that mutations in *SPAST* lead to disease pathogenesis by a haploinsufficiency mechanism [14], [24]–[25]. Alternatively, at least some SPG4 cases appear to involve a dominant-negative mutation mechanism [26]–[27]. Combined, the evidence suggests that precise or stoichiometric levels of spastin are necessary for biological function, which may not be surprising given that spastin forms a hexameric ring with pore loops that bind and tug the tubulin C-terminal tails to disrupt tubulin polymeric interactions and sever microtubules [28]–[30]. Therefore, we hypothesize that alterations in the production level of spastin could contribute to the development or progression of disease. In order to address how spastin is produced, we examined regulatory mechanisms involved in the expression of *SPAST* in eutherian mammals, including human. We demonstrate that the transcription factors (TFs) NRF1 and SOX11 as well as the microRNAs (miRNAs) miR-182 and miR-96 are major factors involved in the regulation of *SPAST*. These findings provide a foundation for understanding regulation of spastin expression, identify new target sequences for mutation screening at the SPG4 locus, and suggest novel approaches to consider for therapeutic approaches in dominantly inherited spastic paraplegia.

## Results

**Figure 1:**
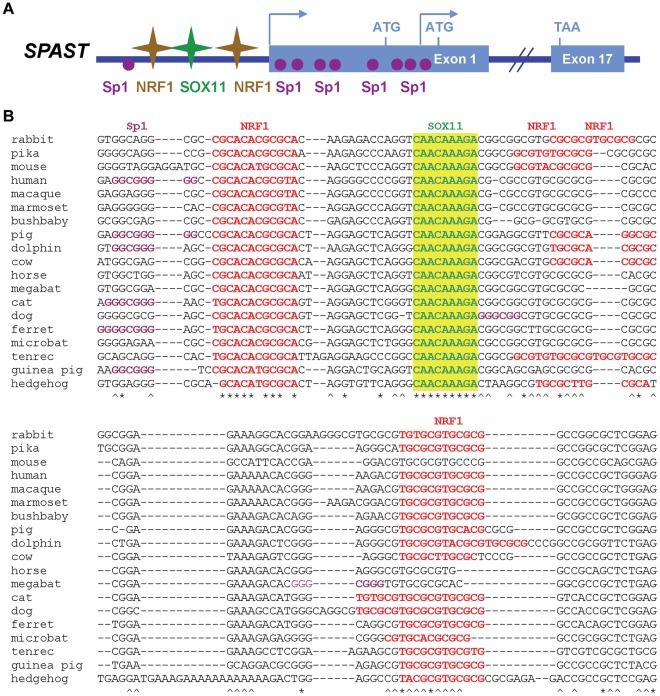
Transcriptional regulation of the *SPAST* gene encoding spastin (SPG4). A ) Cartoon showing the human *SPAST* promoter structure with *cis*-elements representing putative transcription factor (TF) binding sites for NRF1, SOX11, and Sp1. As is typical of CpG-promoters, transcription start sites (TSS) are spread over a large region in exon 1, with two major TSS positions indicated by arrows [31]. There are two alternative translational initiation codons for spastin 68 and 60 kDa polypeptide isoforms, respectively [31]. **B**) Multi-sequence alignment of conserved TF *cis*-elements in representative mammalian species. Sequences were aligned using ClustalW 2.1 and manually adjusted as needed for maximum parsimony. Evolutionarily conserved TF motifs are indicated; *, nucleotide positions conserved in all 19 species; ∧, nucleotide positions conserved in 17/19 species; yellow shading, highly conserved SOX11 motif; red, NRF1 motifs; purple, Sp1 motifs. The NRF1 and SOX11 motifs are highly conserved, but only one Sp1 motif near the 5′ TSS is conserved in mammals. Extended alignments of the complete promoter region into exon 1 and including the first translational start codon are shown in **Fig. S1**.

Previous studies have shown that *SPAST* has numerous transcriptional start sites (TSS) that define the first exon, although two major alternative TSS for *SPAST* can be delineated (**Fig. 1A**) [31]. Interestingly, the region between the two TSS has partial promoter activity [31], while the 179-bp region 5′ of the first TSS has the highest levels of *SPAST* promoter activity [31], [32]. However, the TFs regulating *SPAST* promoter activity have not been previously defined, particularly upstream of the 5′-most TSS. To examine the transcriptional as well as post-transcriptional regulation of *SPAST*, we used molecular phylogenetic methods [33]–[34] to identify conserved sequences of putative TF binding sites and miRNA targeting motifs in the *SPAST* promoter and 3′-UTR, respectively. Subsequently, we used a variety of molecular experimental methods to confirm and extend the predictions from the phylogenetic approach.

### Evolutionary Conserved *cis*-binding Motifs in the *SPAST* Promoter

Using bioinformatics analysis, the non-coding region upstream of the two TSS for *SPAST* was found to be rather poorly conserved in 21 mammals with sufficient sequence coverage for full analysis, without a single motif of ≥6-nt as expected for a TF binding site (**Fig. 1A,B**; **Fig. S1A,B,C**). Nevertheless, several patches of increased conservation corresponded to motifs identical to the consensus binding sites for three TFs. The most highly conserved motif, 5′-CAACAAAGA-3′ (**Fig. 1B**; **Fig. S1A,C**) corresponds to a consensus binding motif for SOX11 and SOX4 [35]–[36], members of the SOX-C family of TFs [37]. This putative TF binding site is identical in 19 of 20 eutherian mammals (**Fig. 1B**) and in two marsupials, the South American opossum (**Fig. S1C**) and tammar wallaby (data not shown), but has been replaced in the elephant (**Fig. S1B**). Flanking either side of the putative SOX11/SOX4 motif were 1–3 copies of a related sequence in different mammals (**Fig. 1A,B**; **Fig. S1A,B,C**), each matching the binding motif 5′-yGCGCAnGCGCr-3′ that is specific for nuclear respiratory factor-1 (NRF1) [38]–[39]. The NRF1 consensus is both a palindrome and repeating pyrimidine (y)-purine (r) sequence, where a maximum of one mismatch in one GCGC motif is allowed to maintain binding affinity [39]–[40], with the 3′-most motif in the *SPAST* promoter a perfect match to the NRF1 consensus. Interestingly, there are four NRF1 motifs in the elephant *SPAST* promoter with 3 of these identical to the consensus and derived by 18-nt and 29-nt duplications that replace the putative SOX11/SOX4 motif (**Fig. S1B**). Finally, although not generally conserved in the same position in all species, there are up to 6 potential binding sites in each species that match the GC-rich consensus for Sp1, typically found in CpG-rich mammalian promoters [41]. Taken together, the phylogenetic analysis suggests that NRF1, SOX11, and Sp1 may contribute to *SPAST* transcriptional regulation in mammals.

### NRF1 Positively Regulates Transcription of *SPAST*

**Figure 2:**
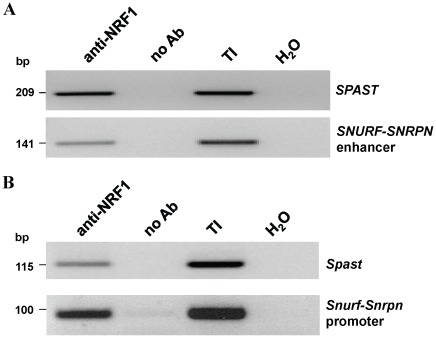
NRF1 chromatin immunoprecipitation (ChIP) assays. NRF1 interacts with the *SPAST* promoter in **A)** human SK-N-SH cells, and **B)** murine Neuro2a cells, by ChIP assay. The *SNURF*-*SNRPN* enhancer for human and *Snurf*-*Snrpn* promoter for mouse are positive controls for *cis*-elements known to be bound by NRF1 [89]. Chromatin was immunoprecipitated with anti-NRF1 antibodies, and the promoter regions were assessed by PCR. Controls are no antibody (no Ab) and total input DNA (TI) for ChIP, and a water control (H_2_O) for PCR.

**Figure 3:**
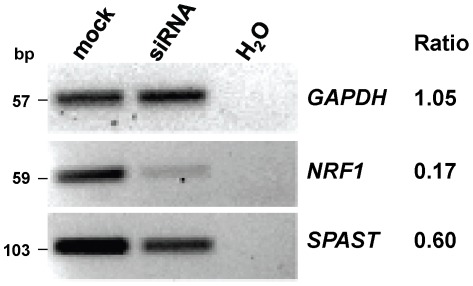
siRNA targeting *NRF1* mRNA knocks down *SPAST* **mRNA expression.** SK-N-SH cells were transfected with a pSUPER-*NRF1* shRNA expression vector or mock transfected, with qualitative analysis of gene expression including a negative control (*GAPDH*). The quantitative band intensities for the “siRNA” and “mock” transfection samples were compared and the ratio is listed to the right of the gel images. The *GAPDH* mRNA level is unaffected by siRNA targeting *NRF1*, whereas the mRNA levels for *NRF1* and *SPAST* are reduced by the siRNA treatment. The experiment was repeated three times with equivalent results to the representative results shown here.

To determine if NRF1 regulates the expression of *SPAST*, we sought to first show that NRF1 physically binds to the *SPAST* promoter. Using chromatin immunoprecipitation (ChIP) assays, we found that NRF1 binds specifically and robustly to the *SPAST* promoter in human SK-N-SH cells (**Fig. 2A**) and in murine Neuro2a cells (**Fig. 2B**). Independently, these data have been validated by ENCODE genome-wide ChIP studies that also demonstrates NRF1 binding to the *SPAST* promoter region (http://genome.ucsc.edu/cgi-bin/hgTracks). To confirm that NRF1 not only binds the *SPAST* promoter but also plays a role in its expression, we determined the effect on *SPAST* expression resulting from reduction of NRF1 levels by targeting *NRF1* mRNA with a specific siRNA. A *NRF1* shRNA expression vector (pSUPER-NRF1) [42] transfected into SK-N-SH cells significantly reduced *NRF1* and *SPAST* transcript levels to<20% and ∼60% of mRNA levels measured in mock-transfected control cells, respectively (**Fig. 3**).

**Figure 4:**
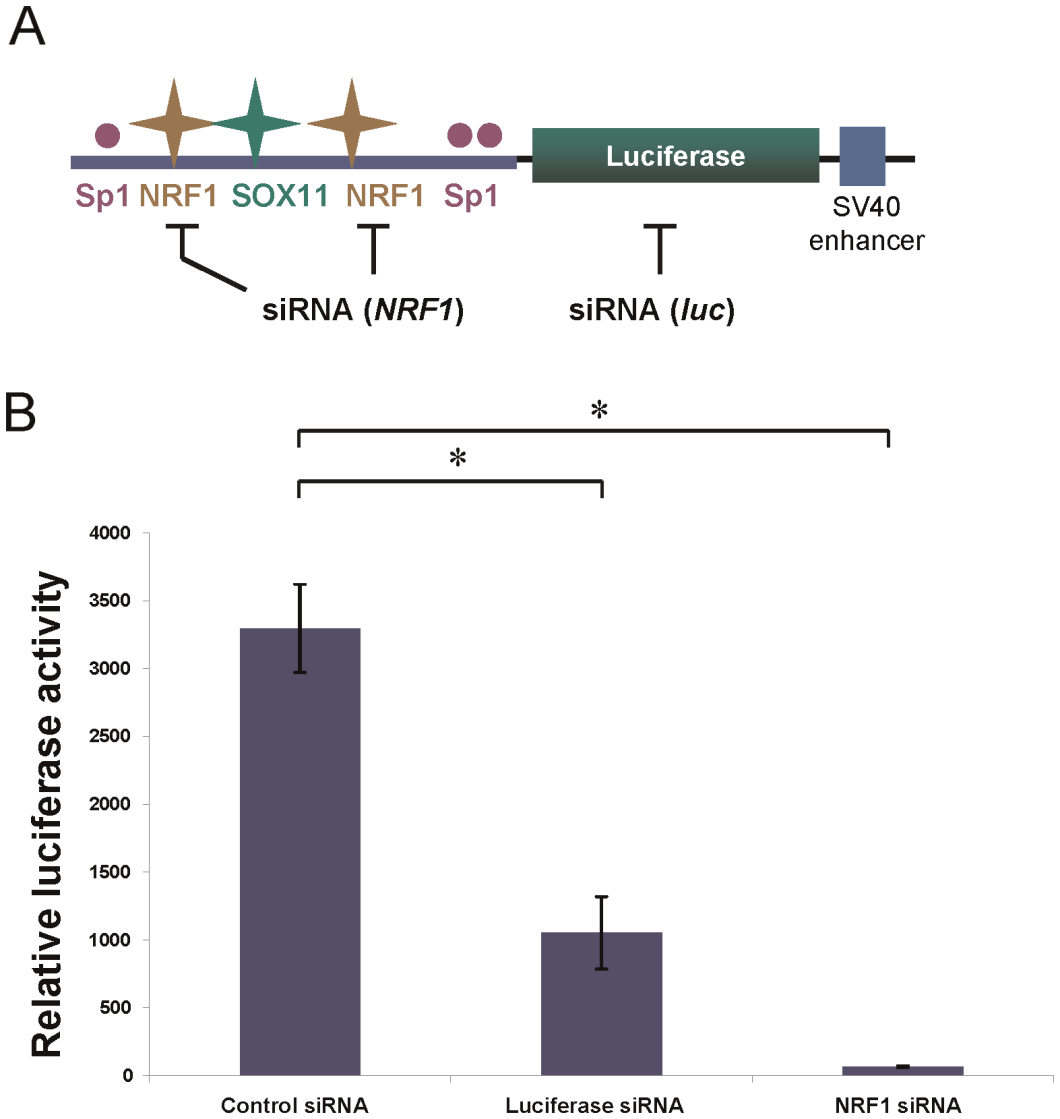
siRNA targeting *NRF1* mRNA ablates *SPAST* promoter function. **A**) Cartoon showing the structure of the pGL3e-SPAST-promoter-luciferase vector, and the inhibitory mechanisms of siRNA action. Symbols are as for **Fig. 1A**. **B**) The plasmid pGL3e-SPAST-promoter was co-transfected into SK-N-SH cells with pSUPER shRNA vectors that target either *NRF1*, luciferase, or negative control (*Arl2*). *, *P*<0.05.

To further validate the role of NRF1 in *SPAST* expression, we generated a *SPAST*-promoter-luciferase reporter construct by ligating a 304-bp fragment of the *SPAST* promoter (-197 to+107 relative to the 5′-TSS) and containing all conserved TF motifs into the pGL3e luciferase reporter vector (**Fig. 4A**). This full-length *SPAST*-promoter construct was then co-transfected into SK-N-SH cells with each of three different pSUPER-shRNA vectors. Compared to control cells expressing an unrelated siRNA [42], which have a high level of luciferase activity, siRNA specially targeting the *luc*-coding sequence significantly decreased luciferase levels by ∼66% (**Fig. 4B**); retention of some luciferase activity is likely due to protein stability or incomplete mRNA knockdown. In contrast, siRNA specifically targeting *NRF1* virtually abolished luciferase activity with only basal levels detected (**Fig. 4B**). Combined, these results show that NRF1 is an essential positive regulator of *SPAST* transcription.

### SOX11 Activates *SPAST* Transcription

**Figure 5:**
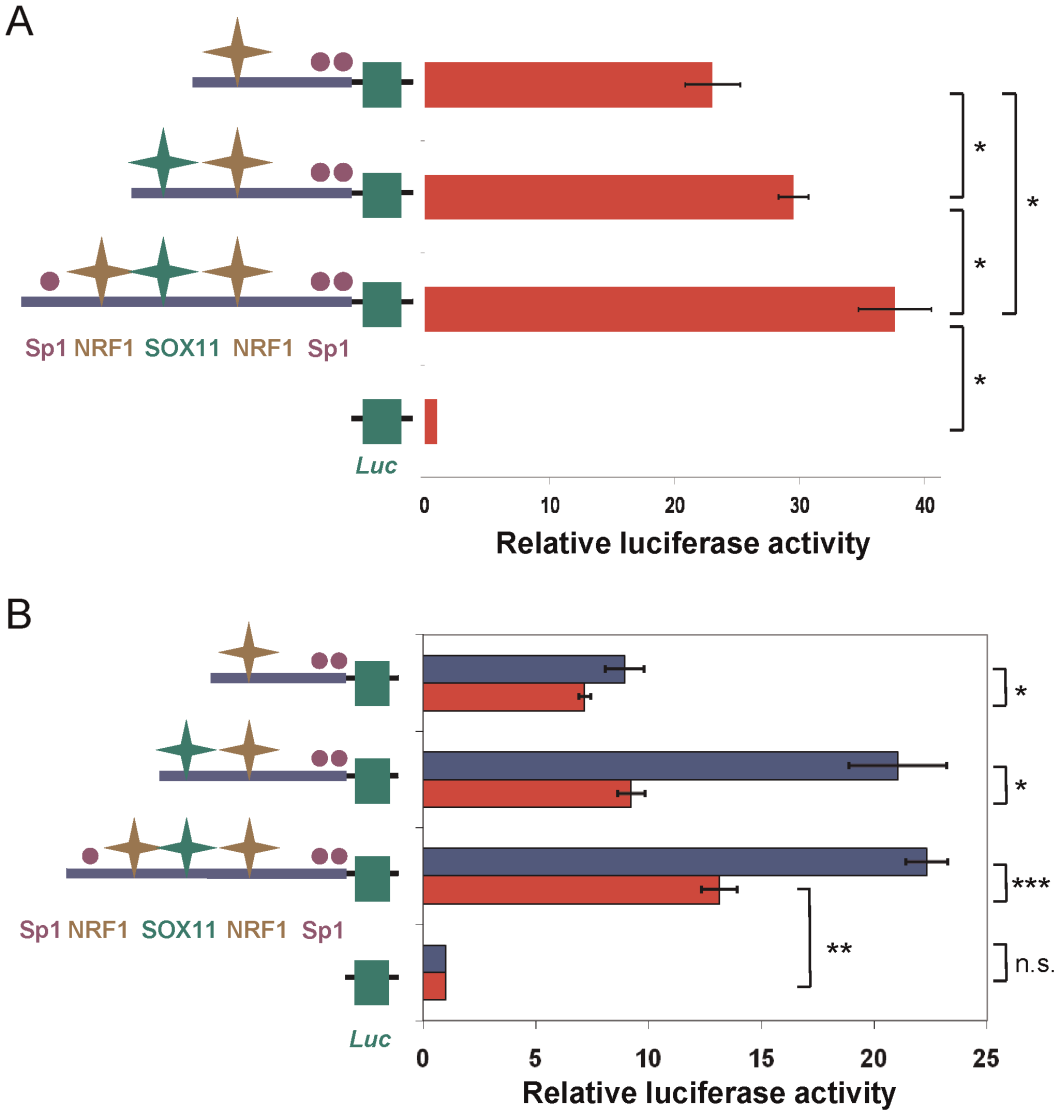
Regulation of the human *SPAST* promoter by SOX11. **A**) Luciferase reporter assays with the human *SPAST* promoter. The plasmid pGL3b-SPAST-promoter-Luc (third row) and two deletion derivatives (depicted in the panel on the left) were transfected into Flp-in293 cells and normalized to cells transfected with the pGL3b vector (fourth row). *, *P*<0.05. **B**) Over-expression of SOX11 upregulates *SPAST* promoter-reporter constructs having a SOX11 binding site. Flp-In-293 cells were transfected with *SPAST*-promoter-luciferase reporter constructs both with (blue) and without (red) the SOX11 expression vector. *, *P*<0.05; **, *P*<0.001; ***, *P*<0.0001, and n.s., not statistically significant. Symbols in A and B are as for **Fig. 1A**.

To determine if the putative SOX11 binding site is involved in transcriptional regulation of *SPAST*, we engineered a set of nested deletions in the full-length *SPAST*-promoter-luciferase reporter construct, which contains a binding site for NRF1 and putative binding motifs for Sp1 and SOX11 (**Fig. 5A**, left). Each construct was transfected into human Flp-In-293 cells. Compared to the full-length *SPAST*-promoter with robust transcriptional activation of the luciferase reporter (almost 40-fold over the pGL3b vector control), deletion of the 5′-most Sp1 motif and an NRF1-like motif (the latter expected to have low affinity in human, due to two changes from the NRF1 consensus [40]) led to a statistically significant ∼20% reduction in luciferase activity (**Fig. 5A**, right). An additional deletion that specifically removed 37-nt including only the putative SOX11 binding motif further significantly reduced promoter-driven luciferase activity by another ∼23% (**Fig. 5A**, right). To establish whether SOX11 binding is responsible for the latter positively-acting *cis*-element, we used co-transfection of a SOX11 expression vector [43] to over-express SOX11 along with the same set of three *SPAST*-promoter-luciferase reporters (**Fig. 5B**, left). Indeed, SOX11 over-expression significantly increased luciferase activity for the two *SPAST*-promoter reporter vectors with the putative SOX11 binding motif but not for the reporter construct that lacked this site (**Fig. 5B**, right). These results indicate that SOX11 acts as a positively-acting TF for *SPAST* expression.

### NRF1 and SOX11 Increase Endogenous Levels of *SPAST *mRNA

**Figure 6:**
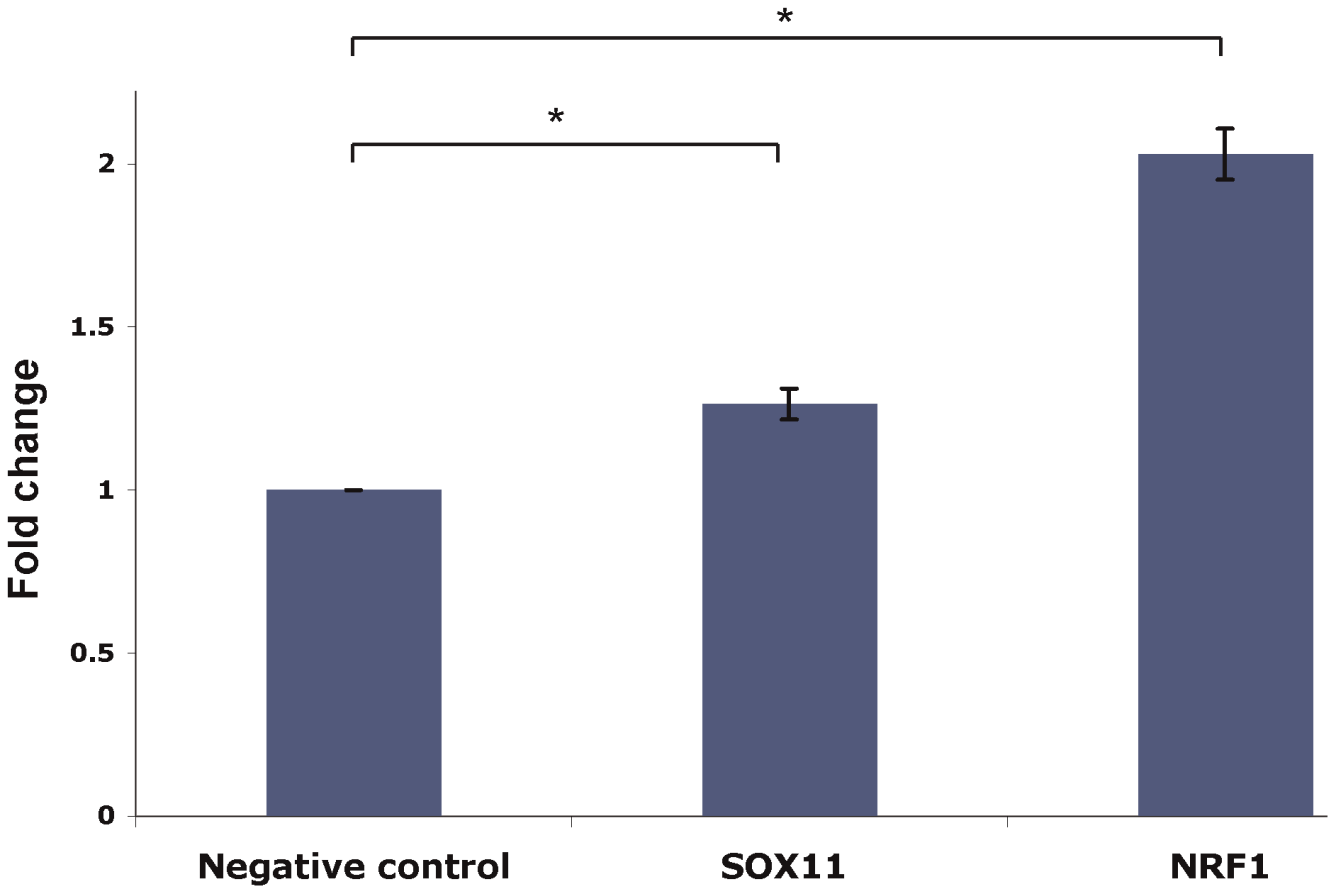
Over-expression of SOX11 and NRF1 upregulates endogenous *SPAST* expression. Cells were transfected with SOX11 or NRF1 expression vectors and compared to cells transfected with the transfection control (pGL3b). By qRT-PCR, with normalization to *GAPDH* mRNA levels and to the transfection control, the levels of *NRF1* and *SOX11* mRNA were increased 182±28-fold and 2,925±241-fold on transfection with NRF1-VP16 and CMV1-SOX11, respectively (data not shown). Similarly, by qRT-PCR analysis, *SPAST* transcript levels are significantly increased by over-expression of SOX11 and NRF1. These data represent the average of three biological replicates each done in triplicate. *, *P*<0.05.

To validate that NRF1 and SOX11 can positively regulate *SPAST* expression in a native chromatin configuration and cellular milieu, we investigated whether these TFs affect endogenous levels of *SPAST* mRNA. To accomplish this we transfected Flp-In-293 cells with SOX11 (pSOX11-CMV) and NRF1 (pNRF1-VP16) expression vectors, as well as an empty plasmid (pGL3b) transfection control. The VP16 transactivation domain fused to NRF1 increases the upregulation of NRF1-target genes by 4-fold compared to NRF1 alone but does not change the specificity [44]. *SPAST* mRNA expression levels were assayed by quantitative qRT-PCR, normalizing 1) to *GAPDH* mRNA level, a gene that is not regulated by NRF1 or SOX11, and 2) to the *SPAST* mRNA level in the pGL3b transfection control. Over-expression of SOX11 or NRF1 led to a significant increase in level of endogenous *SPAST* mRNA (**Fig. 6**).

### A Minimal Role for Elk1 in Regulation of *SPAST*

A recent report suggested that another TF, Elk1, repressed transcriptional activity through a binding site located in the 5′ *SPAST* promoter [32]. However, the putative Elk1 site [32] is poorly conserved in mammals, other than for simian primates (**Fig. S1A**). We tested the potential transcriptional activity of a 182-bp segment upstream of the full-length *SPAST* promoter, lacking conserved sequence elements but containing the putative Elk1 site, using luciferase reporter assays in Flp-In-293 cells. The 182-bp fragment induced a slight but significant increase in luciferase activity over the promoterless control vector whereas when placed upstream of the highly active, full-length *SPAST* promoter there was a slight but non-significant decrease in luciferase activity (**Fig. S2**). These results suggest that Elk1 has a limited role in transcriptional regulation of *SPAST*.

### Evolutionary Conserved *cis*-targeting Motifs in the *SPAST* 3′-UTR

To more fully understand *SPAST* regulation, beyond transcriptional controls, it is necessary to explore mechanisms of post-transcriptional regulation of *SPAST* by miRNAs. Three criteria were assessed to identify candidate miRNA regulators, including 1) presence of optimal 7mer or 8mer seeds [45] and 2) conserved seeds for miRNA targeting within the *SPAST* 3′-UTR, using the TargetScan database, and 3) evolutionary conservation of optimal seeds amongst animals with sequenced genomes, using the BLAST algorithm. Five sets of miRNA seeds match these criteria: miR-96, miR-200bc/429, miR-132/212, miR-30abcde/384, and miR-29abc (**Table S1**). However, only the *SPAST* 3′-UTR seed match for miR-96 was conserved in all mammals (eutherians and marsupials) and in sequenced tetrapod genomes (see below; **Table S1**). We therefore focused on experimental assessment of the role of miR-96 in *SPAST* regulation.

### Post-transcriptional Regulation of *SPAST* by miR-96/−182 miRNAs

**Figure 7:**
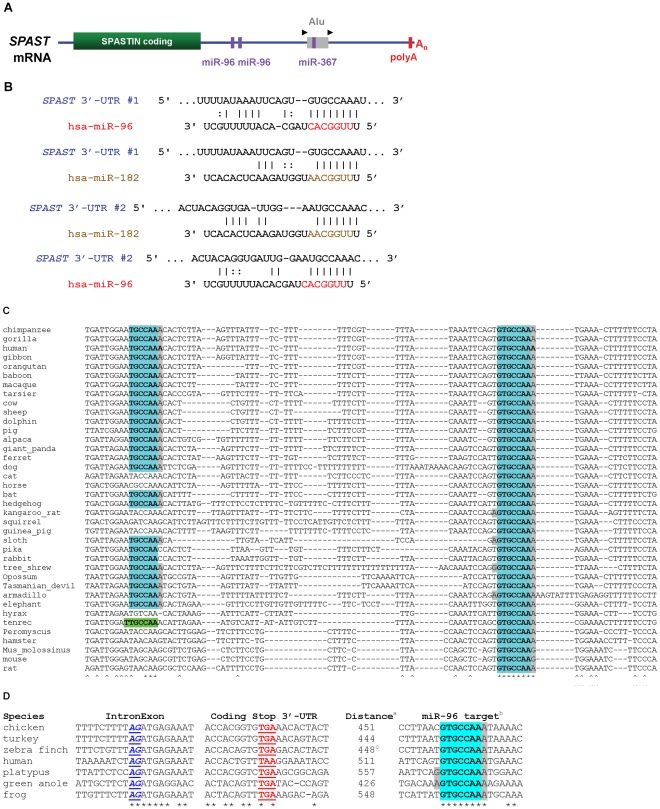
Prediction of several conserved seed motifs for miRNA targets in the *SPAST* 3′-UTR. A ) Cartoon of the *SPAST* mRNA showing positions of the coding sequence (green), polyA site (red), *Alu* repetitive element (gray) with target site duplication (black triangles), and miRNA target sites (purple) analyzed in this study. **B**) Base-pairing of *Homo sapiens* (hsa) miR-96 and miR-182 miRNAs with the *SPAST* 3′-UTR. The optimal seed motifs (red or brown) for targeting by the miRNAs are at position 511–517 (#1) and 462–467 (#2), respectively, of the *SPAST* 3′-UTR. **C**) Evolutionary conservation of the miR-96 target site in the *SPAST* 3′-UTR of mammals. The optimal target for the miR-96 seed (+2 to+8) is shown in blue, an optimal miR-182 seed in green, and other nucleotides that can base pair with miR-96 are in gray. Sequences conserved in all 38 species are indicated by *, and sequences conserved in 35 of 38 (90%) species by ∧. **D**) Evolutionary conservation of the miR-96 target site in the *SPAST* 3′-UTR of tetrapods. Footnotes: ^a^ Distance in nucleotides from stop codon to the first position in the target site for the miR-96 seed. ^b^ The optimal target for the miR-96 seed (+2 to+8) is shown in blue, other nucleotides that can base pair with miR-96 are in gray. ^c^ This distance corresponds to a manually corrected sequence, to overcome a poor sequence assembly in the database version.

Bioinformatic analysis of the human *SPAST* 3′-UTR sequence predict two miR-96 sites, one with an optimal 8mer seed match (ie., 5-UUUGGCAC-3′, nucleotides 1–8) and an adjacent one located 36 to 61-nt 5′ with a potential 7mer-A1 type seed interaction through nucleotides 1–7 of miR-96 (**Fig. 7A,B**). In addition, miR-182, a related miRNA localized in the same polycistronic cluster [46] is also predicted to potentially target *SPAST* via 7mer-A1 type seed interactions at the same positions (**Fig. 7A,B**). Although a recent study identified three unrelated mRNAs targeted and downregulated by miR-96 via 8mer sites but not affected by miR-182 through 7mer-A1 sites [47], we found this does not apply to *SPAST* perhaps due to differences in cell lines, experimental conditions, or the paired target sites in *SPAST*. This finding clearly indicates the importance of experimental verification of *in silico* predictions. The optimal (8mer) miR-96 target site is conserved in the *SPAST* 3′-UTR of all 38 mammalian species with *SPAST* sequences, including two marsupials (opossum and Tasmanian devil), whereas the second (5′) potential miR-96 site is conserved in only 27 of these species (**Fig. 7C**); nevertheless, a second miR-96 (7mer-A1) site is found within 108 to 157-nt 3′ of the optimal miR-96 site in 5 of 10 mammals lacking the 5′ site (data not shown). Remarkably, the miR-96 optimal seed is present in the same relative position in the *SPAST* 3′-UTR in all tetrapods with sequenced genomes, despite otherwise little homology within the 3′-UTR across tetrapod species (**Fig. 7D**). Due to the shared seed motif with miR-96, for position 1–7, miR-182 is likewise predicted to target the *SPAST* 3′-UTR across tetrapods.

**Figure 8:**
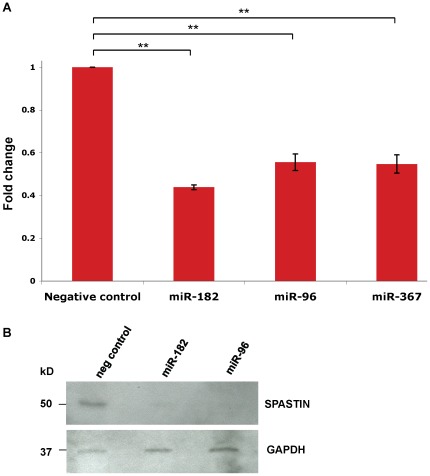
Post-transcriptional regulation of *SPAST.* **A**) QRT-PCR data showing that miR-182, miR-96, and miR-367 reduce *SPAST* transcript levels in Flp-In-293 cells. These data represent the average of three biological replicates each done in triplicate. **, *P*<0.001. **B**) Western blot analysis of spastin and GAPDH protein expression in Flp-In-293 cells transfected with miR-96, miR-182, and the negative (neg) control (see Materials and Methods).

To experimentally demonstrate involvement of miR-96 and miR-182 in the post-transcriptional regulation of endogenous *SPAST* mRNA and/or spastin protein levels, we transfected Flp-In-293 cells with Pre-miRNA oligonucleotides which are then processed in the cytoplasm to the mature miRNAs. Over-expression of miR-96 or miR-182 resulted in statistically significant 45% and 57% decreases in *SPAST* transcript levels, respectively (**Fig. 8A**). By western blotting, over-expression of miR-96 or miR-182 reduced spastin protein to almost undetectable levels (**Fig. 8B**). We conclude that the related miR-96 and miR-182 family of miRNAs provide strong regulation of spastin synthesis in human neural cells and affect both mRNA and protein levels, and that this mechanism likely extends across all tetrapod organisms.

### A Novel miRNA Regulatory Mechanism for *SPAST* in Primates

The human *SPAST* 3′-UTR is unusual in comparison to non-primate animals in having an *Alu* repetitive element insertion in antisense orientation, flanked by an 18-nt target site duplication typical of retrotransposition by the L1 pathway (**Fig. 7A**; **Fig. S3C,D**). Intriguingly, a subset of human miRNAs target sequences within *Alu* elements in the sense or antisense orientation of 3′-UTRs [48]. Among those that target areas of antisense *Alu* elements with minimal sequence variation [48], we focused on the potential targeting of the *SPAST* 3′-UTR *Alu* sequence by the miR-25/32/92ab/363/367 set of miRNAs (**Fig. 7A**; **Fig. S3A,B**). Each miRNA in this set would target the *SPAST* 3′-UTR through an optimal seed of the 7mer-m8 or 8mer type (**Fig. S3B**). We focused on experimental assessment of miR-367, on the basis of the most extensive potential to pair with its potential target (**Fig. S3A,B**), although *in vivo* any of the other miRNAs in this set (miR-25/32/92ab/363) could be involved in *SPAST* regulation. The putative miR-367 target site in the *SPAST* 3′-UTR is present only in catarrhine primates (**Fig. S3C,E**) and correlates with the presence of the *Alu* insertion (**Fig. S3D,E**). Over-expression of miR-367 by transfection of Flp-In-293 cells with Pre-miR-367 resulted in a significant 46% decrease in *SPAST* transcript level (**Fig. 8A**), confirming that miR-367 and/or miRNAs sharing the same seed can regulate *SPAST* expression.

## Discussion

Many biochemical processes contribute to axonal function within the corticospinal tracts, including mitochondrial, endoplasmic reticulum (ER)-shaping, endosomal trafficking, and microtubule stability, each affecting anterograde and retrograde axonal transport [5], [7], [30]. As the longest axons in the body, the corticospinal axons are exquisitely sensitive during human lifespan to mutations in a variety of genes that affect the levels of molecules involved in these functions. This can account for the genetic heterogeneity in HSP [3], [5], [7], although one locus (*SPG4*) accounts for the largest proportion of this genetic load [2], [10], [13]. Based on the genetic characteristics of SPG4, with dominant inheritance and loss of function and/or dominant-negative mechanisms as well as the observed sensitivity of neural cells to modulation of spastin levels [25], we sought to determine the transcriptional and post-transcriptional mechanisms that control *in vivo* production of spastin. By identification of evolutionarily conserved, functional *cis*-elements in the promoter and 3′-UTR of *SPAST*, we have shown that the TFs NRF1 and SOX11 as well as the miRNAs miR-182 and miR-96 play major roles in regulation of spastin levels in neural cells. Together, these findings have implications for understanding regulation of corticospinal neuronal functions, for molecular diagnostic studies in HSP, and for potential therapeutic approaches in neurodegenerative diseases such as HSP.

### Transcriptional Regulatory Mechanisms for *SPAST*

Based on evolutionary conservation and molecular studies, we have shown that NRF1 and SOX11 are the major TFs involved in positive regulation of the mammalian *SPAST* promoter. In addition, the presence of multiple, mostly non-conserved putative Sp1 motifs [this study] in the region between the two major TSS suggests that Sp1 contributes to *SPAST* regulation [31]. Our analysis was limited to promoter regulation of *SPAST* expression using *in vitro* cell models. Future studies will assess *SPAST* regulation *in vivo* and determine if more distant elements such as enhancers, repressors, or boundary elements contribute to *SPAST* regulation.

SOX11 is part of the SOX-C family and is closely related to SOX4 [37], with both TFs sharing *cis*-binding site preferences [35]–[36] and overlapping expression patterns [37]. During neural development, Sox11 and Sox4 are required for neural cell survival but not for lineage specification or differentiation [49]. Both are required for survival of neurons in the developing spinal cord [50]. Sox11 has been shown to be required for survival of sensory neurons [51] whereas in sympathetic ganglia, Sox11 is expressed first and required for proliferation of specific cell types with Sox4 appearing later and required for cell survival [52]. Thus, SOX4 also likely regulates the *SPAST* promoter, as we have shown for SOX11, and both SOX11 and SOX4 may contribute to developmental regulation of *SPAST* gene expression. HMG factors, including SOX11 and SOX4, can bend DNA structures and lead to unwinding or opening of chromatin [53]. Based on these functional properties, we hypothesize that during development SOX11/SOX4 bind the *SPAST* promoter to open chromatin and provide access to other key TFs such as NRF1 that enhance transcriptional activation of *SPAST*.

The SOX11 binding site in the *SPAST* promoter is highly conserved in marsupials and eutherian (placental) mammals with sequenced genomes. Surprisingly, a single sequenced eutherian mammal, the elephant, has lost the SOX11 site which has been replaced by four putative NRF1 binding motifs as a consequence of local DNA duplications. The clustered NRF1 sites in the elephant *SPAST* promoter are similar in structure to *cis*-elements that form enhancer-like elements [54] and suggest cooperative binding with higher levels of transcriptional activation. It is tempting to speculate that this structural arrangement of the *SPAST* promoter in the elephant leads to higher expression levels in the motor cortex and may be associated with greater demands for spastin activity in microtubule severing and/or ER-membrane modeling within the extremely long corticospinal tracts and/or large brain of this species [55].

The close apposition of the SOX11 and NRF1 sites in the *SPAST* promoter suggests that these TFs may interact with each other. Many SOX factors interact with β-catenin and/or TCF (T cell factor) to negatively or positively regulate the canonical Wnt signaling pathway during embryogenesis and neurogenesis [56]. Both SOX11 and SOX4 activate Wnt signaling, and SOX4 binds to both β-catenin and TCF [56]. Intriguingly, recent studies have shown that the *Drosophila* ortholog of NRF1, Erect Wing (EWG), interacts with an Armadillo/β-catenin-TCF complex at specific chromatin sites and promotes Wnt/Wingless signaling in neurons and flight muscle development [57]. Consistent with roles in a common pathway, neurite outgrowth is induced by Wnt signaling [58]–[61], SOX11 [43], [51], [62], NRF1 [63], and spastin [25], [64]. Furthermore, Wnt signaling is involved in guidance of corticospinal axons that descend from the motor cortex and cross the midline into and down the spinal cord [59]. Over-expression of β-catenin decreases microtubule stability [65], consistent with a role in axon growth, branching, and synaptic connections [66]–[67]. Therefore, we hypothesize that the canonical Wnt pathway acts via β-catenin co-activation of SOX11/SOX4 and/or NRF1 to regulate *SPAST* expression.

### Post-transcriptional Regulation of *SPAST* by miRNAs

In this work, we have shown that miR-96 and miR-182 negatively regulate mRNA and protein levels of spastin, with the target sites in the *SPAST* 3′-UTR conserved in all sequenced tetrapod species. Importantly, while miR-96 and miR-182 were previously regarded as specific for sensory neurons [46], [68], recent studies in rodents show higher levels of these two miRNAs in frontal cortex compared to hippocampus [69] and in motor neurons compared to neural stem cells in embryonic spinal cord [70]. Further work is needed to determine whether miR-96/−182 down-regulate *SPAST* levels via target mRNA stability, as with the majority of miRNAs [45], [71]–[72] or whether they can also act through translational regulation. Intriguingly, our data show that another miRNA, miR-367, can also regulate *SPAST* mRNA levels to a degree similar to miR-96 or miR-182, yet the miR-367/25/32/92ab/363 target site in the *SPAST* 3′-UTR only arose in the catarrhine branch of primate evolution coincident with insertion of an *Alu* repetitive element. This gain of novel miRNA regulation targeting *SPAST* correlates with evolutionary changes in anatomy and motor function of the motor cortex and corticospinal tract [73]. Future studies will examine the functional contribution in different neuronal cell types and the evolutionary significance of *SPAST* targeting by each of these miRNAs. In addition, miR-1271 has an identical seed sequence as miR-96 and can function in similar neural pathways [74]. Future studies will determine whether miR-1271 has a role in *SPAST* regulation.

### Implications for Diagnostics and Therapeutics in Neurodegenerative Disease

Identification in this study of evolutionarily conserved, functional motifs in the *SPAST* promoter as *cis*-binding sites for NRF1 and SOX11 has important implications for molecular diagnostic studies in HSP. We propose that mutations in these key *cis*-regulatory elements would reduce spastin levels below a physiological threshold required for axonal function, and hence result in a HSP phenotype. Mutations in TF binding sites in the *SPAST* promoter could occur in either familial HSP or in sporadic spastic paraplegia cases. This mechanism would be consistent with haploinsufficiency as a pathogenic mechanism and, indeed, only slight changes in spastin levels are sufficient to alter neurite stability and growth [25]. NRF1 binding is sensitive to DNA methylation [38], [42], [75], and abnormal epigenetic modifications in the *SPAST* promoter could also perturb *SPAST* expression and contribute to pathogenesis in HSP.

The miRNA regulation of *SPAST* also has implications for mutation studies in neurodegenerative disease. Mutations in miRNA binding sites that prevent pairing between the miRNA and target may upregulate *SPAST* mRNA and protein levels. Given that increases in *SPAST* levels result in neurite phenotypes in a dose-sensitive manner ranging from beneficial to toxic changes [25], dysregulation of *SPAST* levels due to abnormal miRNA dynamics may contribute to pathogenesis of neurological phenotypes.

An understanding of the transcriptional and post-transcriptional regulatory mechanisms controlling expression of *SPAST* and for other HSP loci will determine whether HSP genes are co-regulated in normal neuronal development and aging, and in neurodegenerative conditions such as HSP. These mechanisms may also be relevant to other neurodegenerative conditions, since recent studies have shown that the mRNA levels of several spastic paraplegia gene loci including spastin (spg4), spg7, and spg20, are down-regulated in purified brain neurons from patients with multiple sclerosis (MS), an autoimmune disorder [76]. Indeed, these findings may explain the similar clinical phenotype that occurs with progression of MS, including spastic paraparesis with neurodegeneration in the corticospinal tracts and posterior columns [76].

Knowledge of regulatory mechanisms for spastin synthesis may have therapeutic implications, albeit speculative. As a dominant genetic disorder, SPG4 is dosage-sensitive and thus upregulation of the normal *SPAST* allele could provide therapeutic benefits by preventing or delaying neurodegeneration. For example, NRF1 is positively regulated by AMP-activated protein kinase (AMPK), a cellular energy sensor [77]–[78]. Increased levels or activity of AMPK can be induced by exercise and caloric restriction [79] or with metformin [79]–[80], β-GPA (β-guanadinopropionic acid) [77], resveratol [79], [81], the AMP memetic AICAR (5-aminoimidazole-4-carboxamide ribonucleoside) [81], or novel pharmacological drugs [79], [82]. Indeed, resveratol activates AMPK in neurons and in conjunction with AICAR induces neurite outgrowth [81], and several of these drugs are in use for metabolic syndrome [79] and heart disease [80]. Alternatively, or in combination, targeted knockdown of key miRNA levels in motor cortex neurons could increase spastin expression. These regulatory mechanisms involved in spastin production could also be targeted for cell-based screening to identify small molecule agonists or antagonists, respectively. Treatment by one or more of these approaches could potentially increase NRF1-mediated activation of *SPAST* in association with a haploinsufficient mutation, although success may require that the level of mutant *SPAST* mRNA remain low by nonsense-mediated mRNA decay or proteosomal degradation of unfolded, unstable, or misrouted proteins. These novel ideas for future therapeutic targeting for patients having SPG4 due to haploinsufficient mutations in *SPAST*, and potentially for other neurodegenerative diseases such as MS with downregulation of spastin function, can be tested in animal models [59], [64], [68], [73], [83]–[84] and if and when warranted subsequently in clinical trials.

## Materials and Methods

### Bioinformatics

To identify potential TF binding motifs 5′ of *SPAST* exon 1, we used human *SPAST* genomic sequence and screened for evolutionary conservation among sequenced mammalian genomes [34]. BLAST analyses for sequence similarity (http://blast.ncbi.nlm.nih.gov/Blast.cgi) utilized standard parameters for searches of the non-redundant (NR), high throughput genome sequence (HTGS), and whole genome shotgun (WGS) databases. All extended 5′ sequences spanning the *SPAST* promoter from 21 mammalian genomes are provided in **Dataset S1**. For analysis of the *Alu* insertion in the *SPAST* 3′-UTR, we also used BLAST analysis of primate genomes in the Trace Archives WGS database. When necessary, due to low overall sequence similarity, mammalian and non-mammalian genome sequences for the *SPAST* promoter and 3′-UTR were obtained from the Ensembl genome browser (http://www.ensembl.org/index.html). To identify potential miRNA binding sites in the 3′-UTR of the *SPAST* mRNA, miRNA predictions used TargetScan 5.2 (http://www.targetscan.org/), which is one of two programs shown to most accurately predict *in vivo* miRNA targeting sequences [45], while miRNA sequences are from miRBase (http://www.mirbase.org/). For promoter and 3′-UTR multi-sequence alignments, we used ClustalW 2.1 (http://www.ebi.ac.uk/Tools/clustalw2/index.html). To generate a consensus primate phylogenetic tree for the 10 species of interest, we used version 3 of the *10kTrees* website (http://10ktrees.fas.harvard.edu/) [85].

### Cell Culture

A human neuronal precursor cell line derived from embryonic kidney [86], Flp-In-293 (Invitrogen, Carlsbad, CA), was passaged in Dulbecco’s Modified Eagle Media (DMEM) supplemented with fetal bovine serum (FBS), L-Glutamine, penicillin, streptomycin, and Zeocine (Invitrogen), following the manufacturer’s suggestions. The human neuroblastoma cell line, SK-N-SH (provided by Dr. Nina F. Schor, University of Rochester, Rochester, NY), was passaged in Eagle’s Minimum Essential Media (EMEM) supplemented with FBS, L-Glutamine, penicillin, and streptomycin. The murine neuroblastoma cell line, Neuro2a (provided by Dr. Nina F. Schor, University of Rochester), was passaged in EMEM supplemented with FBS, L-Glutamine, penicillin, streptomycin, and non-essential amino acids.

### Chromatin Immunoprecipitation (ChIP)

Approximately 1×10^6^ cells were plated on 35 cm^2^ plates, and at 70–80% confluency, formaldehyde was added to the media at a final concentration of 1%, and the plates were incubated at 37°C, 10 min to crosslink protein to DNA. Cells were washed twice with 5 ml ice-cold PBS, scraped from the plates using a cell lifter, centrifuged, re-suspended in SDS lysis buffer (ChIP assay kit; Millipore, Billerica, MA), and sonicated to shear DNA. An aliquot of this DNA was taken as a Total Input control. Samples were precleared with protein G-agarose/salmon sperm beads (Millipore), protein-DNA complexes were immunoprecipitated with anti-NRF1 [42], and a no antibody control was similarly processed. Complexes were collected with Protein G agarose/salmon sperm beads and washed, protein-DNA complexes eluted off the beads, and crosslinks were reversed by incubation in NaCl for 16 hr at 65°C. DNA was recovered by phenol-chloroform extraction, precipitated by ethanol, and PCR performed using primers in **Table S2**.

### Expression of siRNA Targeting *NRF1* mRNA

SK-N-SH cells were sub-cultured on 75 cm^2^ flasks for 24 hr prior to nucleofection, and at 80–90% confluence, 4 µg of pSUPER-NRF1 DNA [42], [87] were transfected into 1×10^6^ cells using an Amaxa nucleofector II device (Lonza, Walkerville, MD). The transfected cells were plated on a 6-well plate, and 24 hr later RNA was extracted with Trizol, treated with DNase I to remove genomic DNA contaminants, and 1 µg of RNA from each sample was reverse transcribed into cDNA using SuperScript III reverse transcriptase (Invitrogen) according to the manufacturer’s instruction. PCR was performed using gene-specific primers (**Table S2**) to examine gene expression, with *GAPDH* as a negative control and *NRF1* as a positive control to ensure effective down regulation by siRNA.

### Co-transfections with siRNA and Luciferase Reporter Constructs

The evolutionarily conserved segment of the *SPAST* promoter from -197 to+107 with respect to the *SPAST* 5-TSS (**Fig. 1A**) was isolated by PCR using primers in **Table S1**, and subcloned into the pGL3-enhancer (pGL3e) luciferase vector (Promega, Madison, WI). This pGL3e-*SPAST*-promoter construct was co-transfected into SK-N-SH cells with a shRNA vector targeting *NRF1* (pSUPER-NRF1), or as controls, shRNA vectors targeting luciferase mRNA (pSUPER-*luc*) or an unrelated gene (pSUPER-*Arl2*) [42], and luciferase reporter assays were performed.

### Transcription Factor and miRNA Transfections

For all transfections, cells were seeded in antibiotic-free media the night before transfection. Transfections were carried out in Opti-MEM I (Invitrogen) with Lipofectamine 2000 (Invitrogen) (diluted 1∶100) for 16 hr. For luciferase assays, we seeded 8×10^4^ cells into 24 well plates, and co-transfected Flp-In-293 cells with 1 µg/ml of pGL3b-derived vectors (containing a firefly luciferase gene; Promega) and 25 ng/ml of pRL-SV40 vector (containing a *Renilla* luciferase gene, Promega). To increase cellular levels of miR-96, miR-182, and miR-367, we seeded 7×10^5^ Flp-In-293 cells into 25 cm^2^ tissue culture flasks and transfected them with Pre-miR miR-96, Pre-miR miR-182, Pre-miR miR-367, or Pre-miR Negative Control 1 (Ambion, Austin, TX) at a concentration of a 100 nM. To increase NRF1 and SOX11 levels, Flp-In-293 cells were transfected with 250 ng/ml of pNRF1-VP16 [44] and pCMV-SOX11 [43], respectively. As a negative control for the latter TF experiments, we also transfected Flp-In-293 cells with 250 ng/ml of a non-specific control plasmid (pGL3-basic). All transfections were performed in triplicate.

### Luciferase Reporter Assays

For luciferase assays, we fused various portions of the human *SPAST* promoter to the luciferase gene in pGL3b (Promega). The pGL3b-*SPAST*-promoter construct used the same evolutionarily conserved promoter segment as for pGL3e-*SPAST*-promoter, described above. To remove the 5′ NRF1-like sequence and a Sp1 motif (see **Fig. 1A,B**; **Fig. 5A,B**), we digested pGL3b-*SPAST*-promoter with *Mlu*I and religated the vector, creating pGL3b-*SPAST*-promoter-no-Sp1. To remove the SOX11 binding motif, we digested pGL3b-*SPAST*-promoter-no-Sp1 with *Bss*HII and *Mlu*I and religated the plasmid, creating pGL3b-*SPAST*-promoter-no-Sp1-no-SOX11. In addition, we generated two additional reporter vectors that have a PCR-isolated 182-bp segment (from -379 to -198, with respect to the 5′-TSS) located immediately upstream of the 304-bp *SPAST* promoter segment, to assess activity associated with a putative Elk1 binding site [32] at position -230 to -222 from the 5′-TSS. The 182-bp segment was subcloned by itself in pGL3b as well as upstream of the full-length *SPAST* promoter in pGL3b-*SPAST*-promoter (see **Fig. S2**, left). The *SPAST*-promoter-luciferase derivatives were co-transfected into Flp-In-293 cells with the pRL-SV40 vector containing a *Renilla* luciferase gene, for normalization purposes, and for some experiments, we included pCMV-SOX11. 16 hr after transfection the cells were lysed and luciferase activity was measured using Dual-luciferase reporter assay (Promega) on a Luminometer 20/20^n^ (Turner BioSystems, Sunnyvale, CA). For each sample, the firefly luciferase activity was normalized to *Renilla* luciferase activity, and the relative luciferase activity was normalized to the luciferase activity of cells transfected with pGL3b vector. Each experimental group was done in three biological replicates, and the data were analyzed using a two-tailed t-test.

### Quantitative Gene Expression Analysis

RNA was isolated using the miRNeasy miRNA isolation kit (Qiagen, Valencia, CA). Reverse transcription reactions were performed using the SuperScript III First-Strand Synthesis System for RT-PCR (Invitrogen), following the manufacturer’s suggestions. Quantitative RT-PCR (qRT-PCR) was done on the Applied Biosystems 7300 Real-Time PCR System (Applied Biosystems, Carlsbad, CA) using SYBR Green Master Mix (Applied Biosystems). Primers used for PCR amplification are listed in **Table S2**. Each experimental group was done in three biological replicates, each of which were repeated in three technical replicates, and each qRT-PCR plate included no reverse transcriptase and no template controls. The data were analyzed using the comparative C_T_ method [88]. To examine statistical significance of the data, we analyzed the average of the biological replicates with ANOVA analysis.

### Protein Studies by Western Blot Analysis

Cells were washed with cold PBS and lysed (on ice) with a solution containing 50 mM Tris, 1% Triton X-100, 150 mM NaCl, 1 mM DTT, 10 µg/ml leupeptin, 0.1% sodium dodecyl sulfate, 10 µg/ml pepstatin, and 1 nM phenyl methyl sulfonyl fluoride. Protein concentrations were established using the Bio-Rad Quick Start Bradford Protein Assay Kit (Bio-Rad Laboratories, Hercules, CA). Normalized cell lysates were resolved by sodium dodecyl sulfate polyacrylamide gel electrophoresis (SDS-PAGE). Proteins were transferred to Immobilon-P membranes (Millipore). Membranes were hybridized with an anti-spastin primary antibody (sc-53443; Santa Cruz Biotechnology, Santa Cruz, CA) for 2–4 hr at room temperature. To verify equal protein loading, membranes were stripped with Restore Western Blot Stripping Buffer (Thermo Scientific, Waltham, MA) and probed for GAPDH (Sigma-Aldrich, St. Louis, MO). Membranes were incubated with the corresponding secondary antibody for 1.5 hr at room temperature. Proteins were visualized using the Western Blotting Luminol Reagent (Santa Cruz Biotechnology). Densitometric analyses were carried out using NIH ImageJ software (http://rsbweb.nih.gov/ij/).

## Supporting Information

Dataset S1 ***SPAST*** **promoter-exon 1 sequences from twenty eutherian mammalian and one marsupial species that were used for multisequence alignments in this study.** Symbols are: bold red, consensus motif for NRF1 binding sites; bold green/yellow shade, consensus motif for SOX11 binding site; bold purple, consensus motif for Sp1 binding sites; bold pink, motifs proposed for Elk1 binding sites (see Canbaz et al. 2011), although these are poorly conserved (see **Fig. S1**); bold red and underlined, initiation codon for translation of the 68 kDa spastin isoform; underlined 20–22 nucleotide sequences (human and mouse), ChIP PCR primers; gray shade (human sequence), upstream transcription start site (TSS).
(PDF)

Figure S1 **Evolutionary conservation of the** ***SPAST*** **promoter in mammals identifies transcription factor (TF) binding sites. A)** Multisequence alignment for 19 eutherian mammalian species of the full-length *SPAST* promoter region into exon 1 and including the translational start codon (a segment of 474-nt in human). In addition, *Spast* promoter-exon 1 sequence alignments are shown for **B)** tenrec and elephant, and **C)** western European hedgehog and a marsupial, *Monodelphis domestica* (the tenrec and hedgehog sequences are also shown in **A**). Despite a high degree of nucleotide conservation, elephant is the only sequenced mammalian genome in which the SOX11 site has been lost and replaced by duplications of NRF1 binding site elements (shown by gray shading). Sequences were aligned using ClustalW 2.1 and manually adjusted as needed for maximum parsimony. The TF binding sites identified in this study are indicated in bold type (red, NRF1; green, SOX11; purple, Sp1); a poorly conserved putative site for Elk1 (Canbaz et al. 2011) is also shown (pink); yellow shading, highly conserved SOX11 binding site; INIT, translation start site for spastin; *, nucleotide positions conserved in all aligned sequences;∧, nucleotide positions conserved in 90% (17 of 19) of aligned sequences.
(PDF)

Figure S2 **An extended region upstream of the** ***SPAST*** **promoter that includes a putative Elk1 binding site, has minor effects on transcriptional activity.** A fragment containing the putative Elk1 site (Canbaz et al. 2011) was ligated into the pGL3b vector to generate pGL3b-Elk1 and into the pGL3b-SPAST-promoter construct (the “full-length promoter” construct in **Fig. 5A**) to generate pGL3b-SPAST-promoter-Elk1, which are depicted in the panel on the left. Luciferase assays were performed and the data was normalized to the pGL3b vector, as presented in the panel on the right. Inclusion of the fragment with the Elk1 site led to a significant increase (9-fold) in luciferase activity in pGL3b-Elk1 as compared to pGL3b, but this is significantly less than that from the “full-length promoter” construct with 71-fold increased activity over pGL3b. Inclusion of the fragment with the Elk1 site into the pGL3b-SPAST-promoter construct led to a slight decrease in luciferase activity that was not significantly different than luciferase activity in cells transfected with pGL3b-SPAST-promoter.
(PDF)

Figure S3 **Evolutionary establishment and maintenance of miR-367 targeting of** ***SPAST*** **mRNA in primates. A)** Base-pairing of the *Homo sapiens* (hsa) miR-367 miRNA with the *SPAST* 3′-UTR. The optimal seed motif (green text and yellow shade) for targeting by the miRNA is at position 1,594–1,600 of the *SPAST* 3′-UTR for human, and similarly the optimal seed motif is maintained in chimpanzee, orangutan, and gibbon (see **Fig. S3C**). In macaque and baboon, the *SPAST* target:miR-367 seed pairing includes a U:G pair (see **Fig. S3C**). Additional sequences that can contribute to the *SPAST* target:miR-367 pairing outside the seed are shown by gray shade. **B)** The miRNA seed motif for miR-367 is shared with six other miRNAs, any of which could target the *SPAST* 3′-UTR *in vivo*. At top, the human *SPAST* 3′-UTR sequence that is targeted by miR-367 is displayed, followed underneath by the 7 mature miRNA sequences (from miRBase) that would be capable of targeting the *SPAST* mRNA (yellow shade, miRNA seed and target sequences; gray shade, nucleotides that can contribute to auxiliary pairing, without gaps). **C)** Multisequence alignment in primate species for the boundaries of the Alu element insertion in the *SPAST* 3′-UTR, highlighting the 18-nt target site duplication (TSD; bold blue and underline) and the miR-367 target site (yellow shade, target sequence for optimal miR-367 seed; gray shade, auxiliary pairing). **D)** Multisequence alignment in mammals for the position of the Alu element insertion in the *SPAST* 3′-UTR, highlighting the 18-nt TSD present in catarrhine primates and single copy in the other mammalian species. In **C)** and **D)**: *, conserved in all species;∧, conserved in 13/14 species;+, presence of the Alu element insertion; - (in species name), absence of the Alu element insertion; ##Alu## represents the remaining Alu element sequences, not shown for illustrative purposes; T_n_ represents the number of T nucleotides at that position. **E)** Primate phylogenetic tree showing the presence (+) or absence (-) of an Alu repetitive element insertion in the *SPASTIN* 3′-UTR, that includes a miR-367 optimal seed motif for targeting by the miRNA. An * indicates species in which the pairing of miR-367 with its target includes a G:U pair. The putative origin of the Alu element is shown by a red arrow.
(PDF)

Table S1 **Conservation of** ***SPAST*** **3′-UTR motifs as “optimal seeds” for miRNA targeting.**
(PDF)

Table S2 **PCR primers used in this study.**
(PDF)
