# Supplementary material for: Transcriptional and Post-Transcriptional Regulation of SPAST, the Gene Most Frequently Mutated in Hereditary Spastic Paraplegia
Source: PLoS One. 2012 May 4;7(5):e36505. doi: 10.1371/journal.pone.0036505 (PMC3344893; doi:10.1371/journal.pone.0036505)
Supplement: Dataset S1 — SPAST promoter-exon 1 sequences from twenty eutherian mammalian and one marsupial species that were used for multisequence alignments in this study. Symbols are: bold red, consensus motif for NRF1 binding sites; bold green/yellow shade, consensus motif for SOX11 binding site; bold purple, consensus motif for Sp1 binding sites; bold pink, motifs proposed for Elk1 binding sites (see Canbaz et al. 2011), although these are poorly conserved (see Fig. S1); bold red and underlined, initiation codon for translation of the 68 kDa spastin isoform; underlined 20–22 nucleotide sequences (human and mouse), ChIP PCR primers; gray shade (human sequence), upstream transcription start site (TSS). (PDF) [file pone.0036505.s001.pdf]

Data Set 1. *SPAST* promoter-exon 1 sequences from twenty eutherian mammalian and one marsupial species that were used for multisequence alignments in this study. Symbols are: bold red, consensus motif for NRF1 binding sites; bold green/yellow shade, consensus motif for SOX11 binding site; bold purple, consensus motif for Sp1 binding sites; bold pink, motifs proposed for Elk1 binding sites (see Canbaz et al. 2011), although these are poorly conserved (see **Fig. S1**); bold red and underlined, initiation codon for translation of the 68 kDa spastin isoform; underlined 20-22 nucleotide sequences (human and mouse), ChIP PCR primers; gray shade (human sequence), upstream transcription start site (TSS).

>human
GGCCAATTATCCTTCGGAGAAGACTTAGGTCGCCTGGCAGAAAAAGATGAAAGAAATCTAAGAAAACGACGACACTGAGAGAGGAGCCTAGCG
AACCAGCAGAGCGACCCCAAGCCGCAATTCCCCCTTCCGTGGATCGATTACGAAGG**CTTCCTGGC**AGGAGCTCTCCAGGGCTGCCGACGTGAG
CCGAACTGCACATTGGGAACTGTAGTTGAGTGGGAAAGCCGAGA**GGCGGGGG**C**CGCACACGCGTA**CAGGGGCCCCGGT**CAACAAAGA**CGCGCC
GTGCGCGCGCGCGCCGGAGAAAAACACGGGAAGACG**TGCGCGTGCGCG**GCCGCCGCTGGGAGCCACCAGGCGGCGGAGAGGACAGC**GACAGGA**
AGGGAGG**G**GCCCGAGCCACCGACTGCAGGAGGAGAAGGGGTTGTGCTCCTGGCCGAGGAAGGAGAAA**GGGGCGGGG**CC**GGCGGG**CAGCGTGCG
GCAGTGCGGAGCTCCTGAGACC**GGCGGG**CACACGGGGGTCTGTGG**CCCCCGCC**GTAGCAGTGGCTGCCGCCGTCGCTTGGTTCCCGTCGGTCT
GCGGGA**GGCGGG**TTATGGCGGCGGCGGCAGTGAGAGCTGTGA**ATG**AATTCTCCGGGTGGACGAGGGAAGAAGAAAGGC
>marmoset
GGTCAATTATCATTCGGAGAAGACTTAGGTCGACTGGTAGAAAAAGATGAAAGAAATGTAAGAAAACGATGACATTGAGAAAGGAGCCCTGCG
AACCAGCAGAGCGACCCCAAGCTGCAATTCCCCCTTCGGTGGATCGATTACCAAGG**CTTCCTGGC**AGTACCTCTTCGGGGCTGCTGACGTGAG
CTGAACTGCACATTGGGAATTGTAGTCGAGCCAGGGAAGGAGGAGGGGGGCACCGCACACGCGTACAGGAGCCCAGGT**CAACAAAGA**CGCGCC
GTGCGCGCGCGCGCCGGAGAAAAACACGGGAAGACGGACG**TGCGCGTGCGCG**GCCGCCGCTGGGAGCCACCAAGCGGCAGAGAGGACAGCGGC
AAGGAGGGAGGGGCCCAATCCACCGACTGCAGGAGGAAGAAGGGGTTGTGCTCCTGGCCGAGGAAGGAGAAA**GGGGCGGGG**CC**GGCGGG**CAGC
GCGCGGCGGTGCGGAGCGCCGGAGGCCGGCGGACA**GGCGGGGG**TCTGTGGCCCTAGCCGTCGCAGTGGCCGCTGCTGTCGCTTGGTTGGTTCC
CGTTGGTCTGCGGGA**GGCGGG**TTATGGCGGCGGCGGCAGTGAGAGCTGTGA**ATG**AATTCTCCGGGTGGACGAGGAAAGAAGAAAGGCTCCGGC
AGC
>macaque
TGAGATGTATCCTTTGGAGAAGACTAAGGTCGCCTGGCAGAAAAAGATGAAAGAAACTAGGAAAATTATGACACTGAGAGAGGAGACTCGCGA
ACCAGCAAAGCGACCCCAATCCGCAATTCCCCCTTCGGTGGATCGATTACCAAGG**CTTCCTGGC**AGGCGCTCTCCAGGGCTGCCGACGTGAGC
CGAACTGCACATTGGGAACTGTAGTTGAGCGGGGAAGGCCGAGAGGAGGGCGCCGCACACGCGTACAGGAGCCCCGGT**CAACAAAGA**CGCGCC
GTGCGCGCGCGCCCCGGAGAAAAACACGGGAAGACG**TGCGCGTGCGCG**GCCGCCGCTGGGAGCCACCAGGCGGCGGAGAGGACAGTGGCAGGA
AGGGAGGGGCCCGAGCCACCGACTGCAGGAGGAGAAGGGGTTGTGCTCCTGGCCGAGGAAGGAGAAA**GGGGCGGGG**CC**GGCGGG**CAGCGCGCG
GCGGTGCGGAGCCCCTGAGACC**GGCGGG**CACACGGGGGTCTGTGGCCCTCGCCGTCGCAGTGGCCGCCGCCGTCGCTTGGTTCCCATCGGTCT
GCGGGA**GGCGGG**TTATGGCGGCGGCGGCAGTGAGAGCTGTGA**ATG**AATTCTCCGGGTGGACGAGGGAAGAAGAAAGGC
>bushbaby
AGATACGCATGATAAGACTGATACGCATTTCTTCTTAGATGGATCAATTATCGTTTGAAGAAGATCTGGGTCCCTTGGCAGAAAATGATGAAA
GAAATGTAAGAAAACAACGACACAAAAATATAGGGCCTGCGACCTAGCTTATCTACCCCAAGCCGCAATTTCCCCCGCGTGGGTTGGGCACCT
AGGTTCCCCCGGGGCAGTCCGGAGGCAAAGAACTCGGCGCAGCGACGGAGAGCTGGGCCGATGGGAGCCGAACTGCACATTGGGAATTGTAGT
TCCTCGGGGA**GGGGGGCGG**CGAGCGGCGAGCGC**CGCACACGCGCA**CGAGAGCCCAGGT**CAACAAAGA**CGGCGGCGTGCGCGCGCCGGAGAAAG
ACACAGGAGAACG**TGCGCGTGCGCG**GCGGCCGCTCGGAGCTATCAGGCGGCTGAGAGGACAGAGGGAGGGAGGGGCTGGAGCCTTCGACAGCT
GGAGAAGGAGACCGGGTTGTGCTCCTGAGCGCGGAAGGAGAAA**GGGGCGGGG**CCGGCGGACAGTGTGTGAAGGTGCCGAGCTCTCGCGGCCGG
CCGGCGCGCGGGCGTGTGTGGCCCTAGCCGTCGCAGTGGCCGTCGCCGTCCTTTGGTCCCCGTCGGTCTGCGGGA**GGCGGG**TTATGGCGGCGG
CGGCAGTGGGAGTTGTGA**ATG**AATTCTCCGGGTGGACGAGGGAAAAAGAAAGGCTCCGGCGGCCCCAGC
>pig
TTCAGATAATAATTCCCAGATTGGGACCCATTACTTCCGCAATGGAACTCATATCAATCATTCTTCGTGGAAGATTGGCAGAAAATGTTGAAA
GAAATATAAGAAAACGAGGTCTCTGCAAGAGAGCCCTGCCGCAGCGGTCCATTTCCTGAGTCGACGTCCTAGGTGCCCTGCTAAGGAGCTCCC
CGGGGCAGCGAAGTTGACTGGCCACCTGTCCCTTAAGACGACGTAGGCTGAAGTGCATATTGGGAAATGTAGTTCCCCGGGGAAGCGATAGCG
A**GGCGGGGG**CC**CGCACACGCGCA**CTAGGAGCTCAGGT**CAACAAAGA**CGGAGGCGTT**CGCGCAGGCGC**CGAGAAAGACACGGGAGGGCG**TGCGC**

GTGCACGCGCGGCCGCCGCTCGGAGTTACCAAGCCGCAGAGAGGACGGAGAAAGGGAGGGGCAGGAGCTACCGACCAAAGGAGGAGGAGAAGG
GGTTGTGCTCCTGACCTAGCGAGGAGAAAGGGGCGGGGCCGTCCAGCTGCGCGCGGAGGTGCAGAGCTCCTGGGACCGGCGGGCGCGCGGGGG
TCTCGTGGCCCTTGCCCTCCCAGTAGCCGCCGCCGTCGCTTGACCGCCGTCGGTCTGCGGAAGGCGGGTTATGGCGGCGGCGGCAGTGGGAGC
TGTGAATGAATTCTCCGGGTGGACGAGGGAAGAAGAAAGGCTCCGGCGGCCCCAGCAGCCCGGTGCCTC
>horse
TTACTTCCGAGATGGAACTCAGATCAATCATCCTTTGCGGAAGACTGACAGAAAATGCTGAAATAAATATAAGAAAACGATGACACTGCAAGA
TAGGACCCCTGAGAACATGCCGAACGACCCCAACCCGCAGTTGTCCCTTCCGTGAGTTCATGACCAAGGCTCCCTGCAAAGGAGCTCTCAGGG
GCAGCTTAGTTGCCAGAGCGACAGGTCCCTGAAGACGTCGTAGACCGAACTGCACATTGGGAATTGTAGTTCTGCGGGGACGGACCTAGTGG
CTGGAGCCGCACACGCGCAATAGGAGCTCAGGTCAACAAAGACGGCGTCGTGCGCGCGCACGCCGGAGAAAGACACGGGAGGGCGTGCGCGTG
GCCGCAGCTCTGAGCTACCAGGCGACTGAGAGGACAGAGAGAGGGAGGGGCAAGCGCCACCGACCGCAGGAGGAGGAGAAGGGGTTGTGCTCC
TGGCCGAGCGAGGAGAAAGAGGCGGGGCCGGCAAGCAGTGCGTGGAGGTGCCGAGCTCCTGGGACCGGCGGGCGCGCGGGGGTCTGTGGCCCT
CGCCGTCCCAGTGGCCGCCGCCGTCGCTTGGTCGCCGTCGGTCTGCGGGGAGGCGGGTTATGGCGGCGGCGGCAGTGGGAGCTGTGAATGAAT
TCTCCGGGAGGACGAGGGAAGAAGAAAGGCTCCGGCGGCGCCAGCAGCCCGGTGCCTCCCAGGCCTCCG
>cat
ACACTGAGAGTGTTTTCATGGTACCCATTACTTTTGAGATGGAACACAGGTGAACCATCCCTTGCGGAAGACTGGCAGAAAATGCTGAAAGAA
ATATAAGAAAACGATGACAGTGCGAGATAGGAGCCCTGCGAACTAGGAGAACGACCCCAAGCCGCAGCTGTACCCGCTGCGTTGAGGAGTTGA
TGACCAAGGTTCCCTGCTAAGCAGTTGCCGGGGACAGCGTTGTCAGTAGAGCGACCAGTCCCTGAGGACAACGTAGCCGAACTGTACCTTGGG
AACAGTAGTCCCGCTGGGAAGGGCCCAAAGGGCGGGAACTGCACACGCGCAGTAGGAGCTCGGGTCAACAAAGACGGCGGCGTGCGCGCGCGC
GCCGGAGAAAGACATGGGTGTGCGTGCGCGTGCGCGGTCACCGCTCGGAGCTACCAGGAGGCTGAGAGGACACCGAGAGGGCGGGGCAGGAGC
TACCGACCGCTGGAGGAGGAAAGGGGTTGTGCTCTTGGCCGAGCGCGGAGAAAGGGGCGGAGCCTGCGAGCAGCGCGTGGAGGTGCCGAGCTC
CTGGGACCGGCGGGCGCGCGGGCGTCTGTGGCCCTCGCCGTCCCAGTGGCCGCCGCCGTCGCTTGGTCGCCGTCGTCTGCGGGAGGCGGGTTA
TGGCGGCGGCGGCAGTGGGAGCTGTGAATGAATTCTCCGGGTGGACGAGGGAAGAAGAAAGGCTCCGGC
>ferret
GGAGACTAGCAGAAAATGCTGAAAGAAATATAAGGGCGATAGCACTGCGAGATAGTAGCCCTGCGAACCCGTCGAGCGAGCCCAAACCGCAGC
TGTCCCCACGGTAAATTGATGACTGAGGTTTTCAGTTTAAGCAACTCTCCCGGGATAGCGTAGTCTGTAGAGCGACGGATCCCCGAGGGTGAC
GTAGCTGAATTGCACCTTGGGAACTGTAGTTCCACACGGAAGAGCCCGAGGGGCGGGAGCTGCACACGCGCACTAGGAGCTCAGGGCAACAAA
GACGGCGGCTTGCGCGCGCGCGCTGGAGAAAGACATGGGCAGGCGTGCGCGTGCGCGGCCACAGCTCGGAGCTACCAGGAGGCTGAGAGGACA
CAGAGCGGGAGGGGCAGGAACCACCGACCGCTGGAGGAGGAAAGGGGTTGTGCTCCTGGCCGAGCGAAGAGAAAGGGGCGGGGCCGGCGAGCA
GCGCGTGGAGGTGCCGAGCTCTCGGAACGGCGGACGCGCGGGCGTCTGCGGCCCTCGCTGTCCCAGTGGCCGCCGCCGTGGCTTGGTCGCCGT
CGGTCTGCGGGAGGCGGGTTATGGCGGCGGCGGCAGTGGGAGCTGTGAATGAATTCTCCGGGTGGACGAGGGAAGAAGAAAGGCTCCGGCGGC
TGCAGCAGC
>dolphin
AACTGCTGTCCTCTCATAATAATTACTAAATTGGGACCCATTACTTCTGTGATGGAACTCAGATCAACCAGCCTTTGCGGAAGACCTGGCAGA
AAATGCTGAAAGAAATATAAGAAAACTATGACACCGTAAGATGGCCCTGAGAAATAGCCGAGCGACCCCAGGCTGCAGCGGTCCCTCCAGTGA
GTTGATAGCCTAAGTTCCCTGCTAAAGAGCTCCCAGGGTCAGCGAAGTCGACTGAGCGACCGGTCCCTATGGCAGACGTAGGCCGAACTGCGC
ACTGGGAAATGTAGTTCCATGGGGAAGGGACGGAGTGGCGGGAGCCGCACACGCGCACTAAGAGCTCAGGGCAACAAAGACGGCGGCGTGTGC
GCACGCGCCTGAGAAAGACTCGGGAGGGCGTGCGCGTACGCGTGCGCGCCCGGCCGCGGTTCTGAGCTACCGAGCGGCTGAGAGGACAGAGAG
GGAGGGGCAGGAGCTACCGACCGCAGGAGGAGGAGAAGGGGTTGTGCTCCTGGCCGAGCAAGGAGAAAGGGGCGGGGCTGGAGAGCAGCGCGC
CGGGGTGCCGAGCTCCTGGGACCGGCGGGCGCGTGGGGGTCTGTGGCCCTTGCCGTCCCAGTGGCCGCCGCCGTCGCTTGGCTGCCGTCGGTC
TGCGGGAGGCGGGTTATGGCGGCGGCGGCAGTGGGAGCTGTGAATGAGTTCTCCGGGTGGACGAGGGAAGAAGAAAGGCTCCGGCGGCCCCAG
CAGCCCGGTGCCTCCCAGGCCTCCGCCCCACTGCCT
>dog
TTTGCGGGAGACGGGCAGAAAATGGTGAATGGACTGTAAGAGCGATGACACCGCGAGCCGGGAGCCCTGAGAACCAGGCAAGCGCGACCCCAA
GGCGCAGCTGTCCCCGCCGAGAGCGGGTGGCCGAGGTCCCCTCTGCCGAGCGGCTCCCGGCGACGGCGCTACCCGTAGAGCGACGGATACCGA
GGACCACGTAGCCGAACTGGACGTTGGGAATTGTAAAAGGCAGCGAAGGGGCGCGAGGGGCGCGAGCCGCACACGCGCAGTAGGAGCTCGGTC
AACAAAGAGGGCGGCGTGCGCGCGCGCGCCGGCGAAAGCCATGGGCAGGCGTGCGCGTGCGCGTGCGCGGCCACCGCTCGGAGCTACCAGGAG
GCTGAGGGGACGCAGAGCGGGAGGGGCAGGAGCCGCCGACCGCAGGAGGAGGAAAGGGGTTGTGCTCCTGGCCGAGCGAGGAGAAAGGGGCGG
GGCCGGCGGGCAGCGCGTGGAGGTGCCGAGCTCCTGGGCCCGGCGGGCGCGCGGGCGTCTGTGGCCCTCGCCGTCCCAGTGGCCGCCGCCGTC
CTTGGTCGCCGTCGGTCTGCGGGAGGCGGGTTATGGCGGCGGCGGCAGTGGGAGCTGTGAATGAGTTCTCCGGGTGGACGAGGGAAGAAGAAA
GGCTCCGGC
>cow
AACTGAGATCAACCATTCCTTATGGAAGACCAGCAGAAAATGCTGAAAGAAATATAAGAAAACGACGACACCCCAAGTCCTACAAACTAGCCG
AGGGACCCCAAGCTGCAGCGGTCCCTTTCTTGAGTTAACGGTCTAGATGCCCTGATACTGAGTTACCAGGGTCAGCGAAATCGATTGAGCTAC
GGGTCCCTAAGGGGTAAGTAGGCCGAACTGCATACTGGAAAATGTAGTTCCGCGAGGGAAGGGAGGAATGGCGAGCGGCGCACACGCGCACAA
GGAGCTCAGGGCAACAAAGACGGCGACGTGCGCGCACGCGCCGGATAAAGAGTCGGGAGGGCTGCGCTTGCGCTCCCGGCCGCCGCTCTGAGC
TACCGAACAGCCAAGAGGACAGAGAGAGGGAGGGGCAGGAGCCACCGACCGCCGGAGGAGAAGGGGTTGTGCTCCTGGACGAGCGAGGAGAAA
GGGGCGGGGCCGGCGAGCAGCGCGCGGAGGTGCACCGCTCCTGGGACCGGGGCCGCCCGGGGGTCTGTGGCCCTTGCCGTCCCAGTGGCTGCC
GCCGTCGCTTGGCCGCCGTCGGTCTGCGGGAGGCGGGTTATGGCGGCGGCGGCAGTGGGAGCTGTGAATGAATTCTCCGGGTGGACGAGGGAA
GAAGAAAGGCTCCGGCGGC
>microbat
GGTACCCACTGCTCTGAGACGGGACTCAGATTAATCATCCTTTGAGACAATACTGGAAGAAATCATTAAAACGATAACACTCGGAGAAAGGAG
CCCTGTGAGCAAGCAGAACGACCTCAAACCTCAGCGGTCCTTCTCTGGGACTCGGGGAACAAGGTCGCCAGCCAAGGAGCTCCCAGGCGCAGC
CAAGTCGGCCGAGCGTCGGCCCTTGACGACGACGTAGAGCCGAACTGCACGTTGGGAACTGTAGTTCCACGGAGAAAGGACCGAGGGGAGAAC

GCCGCACACGCGCACGAGGAGCTCTGGGCAACAAAGACGCCGGCGTGCGCGCGCGCCGGAGAAAGAGAGGGGCGGGCGTGCACGCGCGGCC
GCGGCTCTGAGCTCCTAGAGGCGGCCGAGAGGACAGAGAGAGGGAGGGGCAGAAGCCACCGACCTCAGGAAGGGAGAAGGGGTTGTGCTCTTG
GCCGCACGCAGAGAAAGGGGCGGGGCCGGCGGGCGGGGCGCGAAGGTGCCGAGCTCCCGGGACCGGCCGGCGCGCGGGGCTCTGCGGCCCTCG
CCGTCCCAGTGGCCGCTGCCGTCATTTGGTCGCCGTCGGTGTGCGGGAGGCGGGTTATGGCGGCGGCGGCAGTGGGAGCTGTGGATGAATTCT
CCGGGTGGACGACGGAAGAAGAAAGGCTCCGGCGGCCCCAGCAGCCCGGTGCCTCCCAGGCCTCCGGCC
>megabat
GGGACTCAAGTCAATCATCCTTTGCGGAAGACTGGCAGATAATGCTGAAAGAACTACGTATAAGAAAACGATGACACTCTGAGATAGAAACCC
TACGAAACAGCCGAGCAACCCCAAACAGCAGCCGTCTCTTCTGTGAGTTAATGACTAAAGTTCCCAGCTAAGAAACTCCCAGGGGCGGTTAAA
CCCGCAGAGTGACAGTCCCGGAAGACAACGTAGAGAAGAACTGCACATCGGTAACCGTAGTTCCGCGGGGAAGGGAGAGAGTGGCGGACGCCG
CACACGCGCACTAGGAGCTCAGGGCAACAAAGACGGCGGCGTGCGCGCGCGCCGGAGAAAGACACGGGCGGGTGTGCGCGCACGGCGCCGCTC
TGAGCTATATCAGAGGCGGATGAGAGGACAGACACGGGGGAGGAGCAGGATCCGCGGACCGCTGGAGGAGGAGAAGGGGTTGTGCTCCCGGCC
GTGCGAGGAGAAGGGGGCGGGCCGGCGACCGGCGCGTGAAGAGGCCGAGCTCCCGGGGCCGGCCGGCGCGCGGTAGTCTGTGGCCCTCGCTGT
CCCAGTATCCGCTGCCGTCGCTTGGTCGCCGTCGGTCTGCGGAAGGCGGGTTATGGCGGCGGCGGCAGTGGGAGCTGTGAATGAATTCTCCGG
GTGGACGAGGGAAGAAGAAAGGCTCCGGCGGCCCCAGCAGCCCGGTGCCTCCCAGGCCTCCGCCTCCTT
>rabbit
ATACTCACTGCTCCTGAATTCAGACCAATCAACCTTTGTAATAGACTGAAGTCAACTGGCAGAAAATGATGAAACCATGACACCGAGAGATAG
AAGCGCTGCAAACTTACTAGCAGCCCCAAGCCGCCGTCTCCCTATTCAAGGAGTAATTCCCCAGCTTGCTGGTAGGAGCTCCCAGGGCAAGCG
CCGTCCGCGGAGCTACATGGGAGTCGAAGTGCACCTTGGGAAGTGTAGTTCCACGAGGAAAGGGCCGAGTGGCAGGCGCCGCACACGCGCACA
AGAGACCAGGTCAACAAAGACGGCGGCGTGCGCGCGTGCGCGCGCGGCGGAGAAAGGCACGGAAGGGCGTGCGCGTGTGCGTGCGCGGCCGGC
GCTCGGAGCTTCCTTGGCGCTGAGAGGACGGAGAGAAGGGGGGGTTGGAGCCACCGACCGAAGGAGGAGGAGGAGGAGGAGAAAGGGTTGTGC
TCTTGGCCGAGCGAGGGGCGGGGCCGCGGGCGGCGCGTGCAGGTGCCGAGCTCCTGGGACCCGCCGGCGTGCGGGGGGTCTGTGGCCCTCGCC
GTCGAAGCAGTCGCCGCCGTCGCTGGGTCGCCGTCGGTCTGCGGAAGGCGGGTTATGGCGGCGGCGGCGGCAGTGGGAGCTGTGAATGAATTC
TCCGGGTGGACGAGGGAAGAAGAAAGGCTCCGGCGGCGCCAGCAGCCCGGCGCCTCCCAGGCCTCCGCC
>tenrec
ACTTTTCCTCGCAGGTAATATTTCTAATTCCCAGATTGATACGCATTGCTAGATGGAACTCAAAATAATGATGAAAGAAATAGTAGTGAAGAA
AACGATGACATCGCGGGATTATGAACCCTAAGAGCTAACCTAGGGGCTTCAATCGCACTGCCACTTTCCCCGGACCGGGAGCCAACCACCTGG
GAAACGAGGGAAGTCCTCTGAAAAGACACAGCCCAAAGCGTCTGCGTGCGAACTGCATGCTGGGAACTGCAGTTCCGCGGGGAATGGGCCGTG
CAGCAGGCACTGCACACGCGCATTAGAGGAAGCCCGGCCAACAAAGACGGCGGCGTGTGCGCGTGCGTGCGCCGGAGAAAGCCTCGGAAGAAG
CGTGCGCGTGCGTGGTCGTCGCGCTGCGCTACCAGGCGGCGGAGAGTACAGCGAGAGGGAGGGGCTGGTGTCAGACCGGCCGCTGGAGAAGAG
GTTGTGCTCTTCGCCTCGGAAGGAAGAAGGGGCGGGGCCGGCGGGCCGCGCGTGTGGAGGTGCCGGGCTCTAGGGACCGGCGGGCTCGCGGGC
GTCCGTGGCCGTCGCTTCGGCTGCTGCCGTCGCCTGGTCGCCGTCGGTCTGCAGGAGGCGGGTTATGGCGGCGGCGGCAGTGGGGGCTGTGAA
TGAATTCTCCGGGCGGACGAGAGAAGAAAGGCTCCGGCGGCCGCAGCAGCCCGGTGCCTTCCAGGCCTC
>guinea_pig
ATTTTTCGTTTGAGGGTGACATTTGTCTCTCCGACTGATGTCGCCTCAGGTAATAATTTCCGGATTCACTCATACTGTGATCATTTTTTGCCT
AAGACTGACACAATAAAACACTGATACTGGGAGATAGGACCCACGCCAAGCCGGGCTTCTTCCTTCCAGTACAAGCTTCGCTACTCCGAACCC
CCAGGGCCGAGACGTCAATCACAAACCCCTAGTCCGAGACCCACTCGAGTGAAACTGCAGATCCCTGGGGAAGGGGCAGAGAGAAGGCGGGTC
CGCACATGCGCACAGGACTGCAGGTCAACAAAGACGGCAGCGAGCGCGCGCACGCTGAAGCAGGACGCGGGAGAGCGTGCGCGTGCGCGGCCG
CCGCTCTACGTTACCGGGCATTAGAAAAGGACAGAGGGAGCGAGGGAGGGGCTGGAGCCGTGACCCGGCGGAGAAGGCGCTGTGCTCCCGCCG
ACGGAAGGGCAGGGGGCGGGGCCGGCGGGCGGCGCGTGCAGGTGCCCTGCGGCTGAGCCCCGCGGGGGTCCGTGGCCCTCGCGTGGCAGTGGC
CGCCGCTGAAGCTTGGTCGCCGTCGGTCTGAGGGAGGCAGGTTATGGCGACGGCGGCAGTGGGAGCTGTGAATGAGTTCTCCGGGTGGACGAG
GGAAGAAGAAAGGCTCCGGCGGCCCCAGCAGCCCGGTGCCTCGCAGGCCTCCGCCCCCCTGCCCGGCCC
>pika
TTCAGATCAATCAATCTCTGCAAAAGACGAAAAAGTCAACTGGCAGAAAACGATGAAGCCTTGACACAGAAAGCCTTGCTAACCAGCGCAGTA
ACCCCAATCCGCAACCTTGTCCAGGAATCAAGGGATGACGCCCCCAGGTCGCTGGTAGGAGTTCCCAGGGCAAGAGCCGCGCGGAGTCCCACG
TCTGAAGTGCACATTGGGAAATTGTTATTTCGCGAGCAAAGGGGCGAGGGGCAGGCCGCGCACACGCGCAAAAGAGCCCAAGTCAACAAAGAC
GGCGGCGTGTGCGCGCGCGCGCTGCGGAGAAAGGCACGGAAGGGCATGCGCGTGCGCGGCCGGCGCTCGGAGCTCCTGGGCGCCTGAGAGGAC
GGGCGAGCAGGCGGGGTTGGGGTTACCGACCGCAGGAGGAGGAGGAGGAGGAGGAGAAAGGGTTGTGCTCTCGGCCGAGCGAGGGGCGGGGCC
GCGGGCGGCGCGAGCAGGTGCAGGGCTACCGGGACCCGCCGGCGCGCGAGGGTCCTTGGCCCTCGCCGTCGCAGCAAGTGGCCGCCGTCGCTG
GGTCGCCTTGGGTCTGCGGGAGGCGGGTTATGGCGGCGGCGGCGGCGGCAGTGGAAGCCGTGAATGAATTCTCCGGGTGGACGAGGGAAGAAG
AAAGGCTCC
>mouse
CCTTCCTCAGCTACAAGAGGAGTTGGAGGTCAGCATCTCACACAAAGGCAAACAACCGCACTGCGAGACAGCAGACCTGAGCACTAGTAAAGC
CACTCAAACGCTGTTAACCCCCATTTAAAAATTTATTATCAAAGCTCCCTATTCCGATTCTTCAAAAGTCGAAGAACCTGCGGAGCGACGAAC
CCCTGGGACCATCGCAAGCCGAACCGCGCCTACAGAACTGAGGTTCCCAGAGAAAGGGGCATAGCAGGGGCGTGGGGGGTAGGAGGATGCCGC
ACATGCGCACAAGCTCCCAGGTCAACAAAGACGGCGGCGTACGCGCGCGCACCAGAGCCATTCACCGAGGACGTGCGCGTGCCCGGCCGCCGC
AGCGAGGTGCCAGCGGCCCAGGGGACAGAAGGAAAGGAGGGGCAGGAGCCACCGGCCGCGGCGGGAGGAGGAGGAGGAGGAGGAGACGGG
GTTGTGCTCCTGGGCAGCTGAGGAGCGAGGGGCGGGGCCGGCGAGCGGCGCGTGCAGGTGCCAGGCTCCCGGGCTCCGGCTGGCGCGCGAGCG
GCTCCGTGGGCCCCCGCCGCCGCAGTGGCAGTGGCCGCCGCCGCCGCCGCTTGGTCGCCGTCGGTCTGCGGGAAGCGGGTTATGGCGGCGGCG
GCAGTGGGAGCTGTGAATGAGTTCTCCGGCCGGACGACGGAAGAAGAAAGGCTCGGGCGGCGCGAGCCC
>elephant
TTTCTATATACGGGTTGCTTTTTAGATGGAACTCAGAGCAATCACCCTTTGGAGAAGACTTGAGTCAACTGACAGAAATGACGAAAGAAACGT
TAAGTACAGAAAACCTAAACATTGTGAGATATGAGCCCTGGGAACTAGCCGAGGGACCCTGAGAACAGTCGCCCCTCCCCTGGGCTGATAGCC
AATGTTCCTGGCTATGAAGGAGTCAGCAGCCTCGGTGCCCGAAGTCGGCAGGCTCAGAACTGCACGCTATGACCGGCAGTTACACTGGGAACT

GGCGC**CGCTCACGCGCA**CTAGAACCCAGGTCGACGAAGATGGCGCTTTAGAAAG**CCGCCC**AAGAAATACATGGAAAGACG**TGCGCGTGCGCG**C
CAGTGAAAGACACGAGGGGAGGGGCG**TGCGCGTGCGCG**CCAGGGAGAGACACGGAGGAAGAGACG**TGCGCGTGCGCG**CAGCTGTAGCACTGAG
CCACCAGGCACTCCGGAAGACAGAGGGAGGTAGGGGTGGAGCAACTGCTGGTGTCAGACTCCCGCATGAAAATGGCTTGTTCTTCTGACTGAA
GAAGCC**GGCGGG**CAACGCGTGGAGCTGCCGAGCTCTGGGGCCAGCGGGCGCGCGGGGGTTTGTGGCCATCGCCGTGGCCGCCGCCGTGGCTTG
GTCATCGTGGTTCTGCGGGA**GGCGGG**TTATGGCGGCGGCGGCAGTAGGAGCTGTGG**ATG**AATTCTCCGGGTGGACGACGGAAGAAGAAAGGCT
CCGGCGGCCCCAGCAGCCCGGTGCCTCCCAGGCCTC
>hedgehog
TGGGAGGAGAGGGAGAGAAAGAACCAGATATCACTCTGGTAAAGGTGCTGCTGGGGACTGAACTCAGACAAGAGCCAAGAGACTTAGTTCCGC
AATCTCATCACGCAGTCTTTTGGGAGAAGATTGACACTCCCTGGTAGATCTCAGGCCTTCCCTCCCCCCACCAATGGACGACGTCCTGATATG
ATGTAAAGCGAGCAGCTGTACGCTGGGAATTGTAGTCTGCTGGGAGTAGAACGACCGCAGGTGGAGGGCGCA**GCACATGCGCA**CTAGGTGTTC
AGGG**CAACAAAGA**CTAAGGCG**TGCGCTTGCGCA**TTGAGGATGAAAGAAAAAAAAAAAAAGACTGGAGGCCG**TACGCGTGCGCG**CGCGAGAGACC
GCCGCTCCGAGCTGCCCGGCAGCCGAGGGGACGCGGGTCGGAGGGAGGGGCAGGAGTCGAGCCGGCGGCCGCAGGAGGCGGAGAGGGGTTGCG
CTCTCAGCCGGCCACAGGCGGCAACGAGGAA**GGGGCGGG**GCCGCGCGCCGAGGGGCCGAGCCCCGGGACC**GGCGGGGGG**CGCGGGCCCTCGTC
GTGCCGGGGCCACCACCGCCGCTCGGTGGCCCTGGCTCTCGGGTTATGGCGGCGGCGGCAGCGGGGGCTGTGA**ATG**AATTCTCCCGGCCGACG
AGGAAAGAAGTCAGGCTCCGGCGGCGCCCGCAGCCCGGTGCCT**CCCCGCCGCCC**ACCGGCACGGC
>Monodelphis
GCCTTGCACACAGTAGGTACTTAATAAATGCTAGTTAATTAACCCAGGCAAGTTTACATATTGGAAAAGGAGGATAAAAATAAGATTGTAGTG
AGGTTCAAATTAGATAATGGAAAACTCTTCGTCAATCTTAAAGGACTCATAAACGCCAGTTATCATTAATCGACAAAGCACCAACAAGCAAAG
ATAATTCCGAGTTCAGGCTGGCCCTAAAGGGCTACAAAAGACGCTGGGACTTTCAGCTTCAGAGAAGGCGGAGCCTAGACGAGGT**GGGCGGGG**
ATGAAAGGAAGGC**TGCACAAGCGCA**CCGCGGGAGCTCCTGT**CAACAAAGA**CCGAGGCACGCTCGTGAGCAACGGAGGCTAAGATCCTATGGAA
CGTGCGCGTGTCCGCCTCGCTGAGGCAGCCTATGAAAGGAGAAAGAAAGGACAGGACGTGCGCGCGACTGTCCCATTGCCTGAGCCTATGGCC
GTGAGAGAGGA**GGGCGGGGCGGG**ACCAGGACAGATCCCGGACATCAAAACCTGCTCGGGAGGGTTTCGGAGAGGGGGAGGTGACGCTTCGGAC
CGAGGGAA**GGGGCGGGG**CCGGGTGTGGAGGTAGCTGGAGCTCTGAGGACTCGCCCGACTGCTACCCAGCTTTGGTGCTTTGCCCTTCTCCTCC
TCCTCTTGCCTGCAGCACCAGCACCACCGCCGCTTGGTCGCTGACAGCGGGTTATGGCGGCGGCGGCAGTGGGAGCCGTGA**ATG**AATTCTCCG
GGCGGCCGAGGGAAGAAGAAAGGCTCCGGCACTGCCGCGCCCGCTGGGCCCCC
