# Supplementary material for: Transcriptional and Post-Transcriptional Regulation of SPAST, the Gene Most Frequently Mutated in Hereditary Spastic Paraplegia
Source: PLoS One. 2012 May 4;7(5):e36505. doi: 10.1371/journal.pone.0036505 (PMC3344893; doi:10.1371/journal.pone.0036505)
Supplement: Figure S1 — Evolutionary conservation of the SPAST promoter in mammals identifies transcription factor (TF) binding sites. A) Multisequence alignment for 19 eutherian mammalian species of the full-length SPAST promoter region into exon 1 and including the translational start codon (a segment of 474-nt in human). In addition, Spast promoter-exon 1 sequence alignments are shown for B) tenrec and elephant, and C) western European hedgehog and a marsupial, Monodelphis domestica (the tenrec and hedgehog sequences are also shown in A). Despite a high degree of nucleotide conservation, elephant is the only sequenced mammalian genome in which the SOX11 site has been lost and replaced by duplications of NRF1 binding site elements (shown by gray shading). Sequences were aligned using ClustalW 2.1 and manually adjusted as needed for maximum parsimony. The TF binding sites identified in this study are indicated in bold type (red, NRF1; green, SOX11; purple, Sp1); a poorly conserved putative site for Elk1 (Canbaz et al. 2011) is also shown (pink); yellow shading, highly conserved SOX11 binding site; INIT, translation start site for spastin; *, nucleotide positions conserved in all aligned sequences;∧, nucleotide positions conserved in 90% (17 of 19) of aligned sequences. (PDF) [file pone.0036505.s002.pdf]

Figure S1. Evolutionary conservation of the *SPAST* promoter in mammals identifies putative transcription factor (TF) binding sites. A) Multisequence alignment for 19 eutherian mammalian species of the full-length *SPAST* promoter region into exon 1 and including the first translational start codon (a segment of 474-nt in human). In addition, *Spast* promoter-exon 1 sequence alignments are shown for **B)** tenrec and elephant, and **C)** western European hedgehog and a marsupial, *Monodelphis domestica* (the tenrec and hedgehog sequences are also shown in **A**). Despite a high degree of nucleotide conservation, elephant is the only sequenced mammalian genome in which the SOX11 motif has been lost and replaced by duplications (shown by gray shading) of putative NRF1 binding site elements. Sequences were aligned using ClustalW 2.1 and manually adjusted as needed for maximum parsimony. The putative TF binding sites identified in this study are indicated in bold type (red, NRF1; green, SOX11; purple, Sp1); a poorly conserved putative site for Elk1 (Canbaz et al. 2011) is also shown (pink); yellow shading, highly conserved SOX11 motif; gray shade (human sequence), upstream transcription start site (TSS); INIT, translation start site for the 68 kDa spastin isoform; *, nucleotide positions conserved in all aligned sequences; ^, nucleotide positions conserved in 90% (17 of 19) of aligned sequences.

A

```
                    Elk1
rabbit      TAAT-TCCCCAGC--TTGCTGGT-------------AGGAGCTCCCAGG-GCAA-GCGCCGTCCGCGGAGCTACATGGGA-GTCG--AAGTGCACCTTGG-GAAGT-GTAGTTCCACGAG
pika        TGACGCCCCCAGG--TCGCTGGT-------------AGGAGTTCCCAGG-GCAA-GAGCC---CCTGGGACCAT-CGCAA-GCCG--AACCGCGCCTACA-GAACT-GAGGTTCCCAGAG
mouse       CCCTATTCCGATTCTTCAAAAGTCG-----------AAGAACCTGCGGA-GCGACGAACC---CCTGGGACCAT-CGCAA-GCCG--AACCGCGCCTACA-GAACT-GAGGTTCCCAGAG
human       GAAGGCTTCCT------GGCAG---------------GAGCTCTCC-----------AGG---GCTG---CCG-ACGTGA-GCCG--AACTGCACATTGG-GAACT-GTAGTT----GAG
macaque     CAAGGCTTCCT------GGCAG---------------GCGCTCTCC-----------AGG---GCTG---CCG-ACGTGA-GCCG--AACTGCACATTGG-GAACT-GTAGTT----GAG
marmoset    CAAGGCTTCCT------GGCAG---------------TACCTCTTC-----------GGG---GCTG---CTG-ACGTGA-GCTG--AACTGCACATTGG-GAATT-GTAGTC----GAG
bushbaby    CTAGGTTCCCCC---GGGGCAGTCC---GGAGGCAAAGAACTCGGCGCA-GCGACGGAGA---GCTGG-GCCG-ATGGGA-GCCG--AACTGCACATTGG-GAATT-GTAGTTCCTCGGG
pig         CTAGGTGCCCT--GCTAAGGAGCTC-CCCGGGGCAGCGAAGTTGACTG--GCCACCTGTC---CCTTAAGACG-ACGTAG-GCTG--AAGTGCATATTGG-GAAAT-GTAGTTCCCCGGG
dolphin     CTAAGTTCCCT--GCTAAAGAGCTC-CCAGGGTCAGCGAAGTCGACTGA-GCGACCGGTC---CCTATGGCAG-ACGTAG-GCCG--AACTGCGCACTGG-GAAAT-GTAGTTCCATGGG
cow         CTAGATGCCCT--GATACTGAGTTA-CCAGGGTCAGCGAAATCGATTGA-GCTACGGGTC---CCTAAGGGGT-AAGTAG-GCCG--AACTGCATACTGG-AAAAT-GTAGTTCCGCGAG
horse       CAAGGCTCCCT--GCAAAGGAGCTC-TCAGGGGCAGCTTAGTTGCCAGA-GCGACAGGTC---CCTGAAGACG-TCGTAG-ACCG--AACTGCACATTGG-GAATT-GTAGTTCTGCGGG
megabat     TAAAGTTCCCA--GCTAAGAAACTC-CCAGGGGCGGTTAAACCCGCAGA-GTGACA-GTC---CCGGAAGACA-ACGTAGAGAAG--AACTGCACATCGG-TAACC-GTAGTTCCGCGGG
cat         CAAGGTTCCCT--GCTAAGCAGTTG-CCGGGGACAGCGTTGTCAGTAGA-GCGACCAGTC---CCTGAGGACA-ACGTAG--CCG--AACTGTACCTTGG-GAACA-GTAGTCCCGCTGG
dog         CGAGGTCCCCTCTGCCGAGCGGCTC-CCGGCGACGGCGCTACCCGTAGA-GCGACGGATA---CC-GAGGACC-ACGTAG--CCG--AACTGGACGTTGG-GAATT-GTAAAAG-GCAGC
ferret      TGAGGTTTTCAG-TTTAAGCAACTCTCCCGGGATAGCGTAGTCTGTAGA-GCGACGGATC---CCCGAGGGTG-ACGTAG--CTG--AATTGCACCTTGG-GAACT-GTAGTTCCACACG
microbat    CAAGGTCGCCA--GCCAAGGAGCTC-CCAGGCGCAGCCAAGTCGGCCGA-GCGTCG-GCC---CTTGACGACG-ACGTAGAGCCG--AACTGCACGTTGG-GAACT-GTAGTTCCACGGA
tenrec      CAA--CCACCT-----GGGAAACGA----------GGGAAGTCCTCTGAAAAGACACAGC---CCAAAGCGTCTGCGTG----CG--AACTGCATGCTGG-GAACT-GCAGTTCCGCGGG
guinea pig  CAAGCTTCGCT---------ACTCC-------------GAACCCCCAGGGCCGAGACGTCAATCACAAACCCC----TAG-TCCG--AGACCCACTCGAGTGAAACTGCAGATCCCTGGG
hedgehog    TGACACTCCCT------GGTAGATCTCAGGCCTTCCCTCCCCCCACCAATGGACGACGTC----CTGA-TATG-ATGTAAAGCGAGCAGCTGTACGCTGG-GAATT-GTAGTCTGCTGGG
              ^     ^^                                                                  ^       ^  *^  ^       ^ ^**   * ^^^       ^

                                       Sp1             NRF1                     SOX11           NRF1   NRF1
rabbit      GAAAGGG------------CCGAGTGGCAGG----CGC-CGCACACGCGCAC---AAGAGACCAGGTCAACAAAGACGGCGGCGTGCGCGCGTGCGCGCGCGGCGGA------------G
pika        CAAAGGG------------GCGAGGGGCAGG----CCG-CGCACACGCGCAA---AAGAGCCCAAGTCAACAAAGACGGCGGCGTGTGCGCG--CGCGCGCTGCGGA------------G
mouse       AAAGGGGCATAGCAGGGGCGTGGGGGGTAGGAGGATGC-CGCACATGCGCAC---AAGCTCCCAGGTCAACAAAGACGGCGGCGTACGCGCG----CGCAC--CAGA------------G
human       T-GGGAAAG----------CCGAGAGGCGGG----GGC-CGCACACGCGTAC---AGGGGCCCCGGTCAACAAAGACG-CGCCGTGCGCGCG----CGCGC--CGGA------------G
macaque     C-GGGGAAGG---------CCGAGAGGAGGG----CGC-CGCACACGCGTAC---AGGAGCCCCGGTCAACAAAGACG-CGCCGTGCGCGCG----CGCCC--CGGA------------G
marmoset    CCAGGGAAG-----------GAGGAGGGGGG----CAC-CGCACACGCGTAC---AGGAGCCCAGGTCAACAAAGACG-CGCCGTGCGCGCG----CGCGC--CGGA------------G
bushbaby    GAGGGGGGCG---------GCGAGCGGCGAG----CGC-CGCACACGCGCAC---GAGAGCCCAGGTCAACAAAGACG---GCG-GCGTGCG----CGCGC--CGGA------------G
pig         GAAGCGAT------------AGCGAGGCGGG----GGCCCGCACACGCGCACT--AGGAGCTCAGGTCAACAAAGACGGAGGCGTTCGCGCA----GGCGC--C-GA------------G
dolphin     GAAGGGAC------------GGAGTGGCGGG----AGC-CGCACACGCGCACT--AAGAGCTCAGGGCAACAAAGACGGCGGCGTGTGCGCA----CGCGC--CTGA------------G
cow         GGAAGGGA------------GGAATGGCGAG----CGG-CGCACACGCGCACA--AGGAGCTCAGGGCAACAAAGACGGCGACGTGCGCGCA----CGCGC--CGGA------------T
horse       GACGGGAC------------CTAGTGGCTGG----AGC-CGCACACGCGCAAT--AGGAGCTCAGGTCAACAAAGACGGCGTCGTGCGCGCG----CACGC--CGGA------------G
megabat     GAAGGGAG------------AGAGTGGCGGA----CGC-CGCACACGCGCACT--AGGAGCTCAGGGCAACAAAGACGGCGGCGTGCGCGC------GCGC--CGGA------------G
cat         GAAGGGCC------------CAAAGGGCGGG----AAC-TGCACACGCGCAGT--AGGAGCTCGGGTCAACAAAGACGGCGGCGTGCGCGCG----CGCGC--CGGA------------G
dog         GAAGGGGCG-----------CGAGGGGCGCG----AGC-CGCACACGCGCAGT--AGGAGCTCGG-TCAACAAAGAGGGCGGCGTGCGCGCG----CGCGC--CGGC------------G
ferret      GAAGAGCC------------CGAGGGGCGGG----AGC-TGCACACGCGCACT--AGGAGCTCAGGGCAACAAAGACGGCGGCTTGCGCGCG----CGCGC--TGGA------------G
microbat    GAAAGGAC------------CGAGGGGAGAA----CGC-CGCACACGCGCACG--AGGAGCTCTGGGCAACAAAGACGCCGGCGTGCGCGCG----CGCGC--CGGA------------G
tenrec      GAATGGGC------------CGTGCAGCAGG----CAC-TGCACACGCGCATTAGAGGAAGCCCGGCCAACAAAGACGGCGGCGTGTGCGCGTGCGTGCGC--CGGA------------G
guinea pig  GAAGGGGCAG---------AGAGAAGGCGGG------TCCGCACATGCGCAC---AGGACTGCAGGTCAACAAAGACGGCAGCGAGCGCGCG----CACGC--TGAA------------G
hedgehog    AGTAGAACGAC-------CGCAGGTGGAGGG----CGCA-GCACATGCGCACT--AGGTGTTCAGGGCAACAAAGACTAAGGCGTGCGCTTG----CGCATTGAGGATGAAAGAAAAAAA
                                     ^*   ^         ***** *** *    ^ *    * ^^ *********^^  ^ *^^^ *^^^      ^* ^    ^^            ^
```

```
                                      NRF1                                                                           TSS
rabbit      AAAGGCACGGAAGGGCGTGCGCGTGTGCGTGCGCG----------GCCGGCGCTCGGAGCTTCCT---TGGCGC-TGAGA-GGACGG----A-GA--GAAGGGGGGGTTGGAGCCA--CC
pika        AAAGGCACGGA------AGGGCATGCGCGTGCGCG----------GCCGGCGCTCGGAGCT-CCT---GGGCGCCTGAGA-GGACGG----GCGA--GCAGGCGGGGTTGGGGTTA--CC
mouse       CCATTCACCGA-------GGACGTGCGCGTGCCCG----------GCCGCCGCAGCGAGGTGCC-----AGCGGCCCAGG-GGACAG----AAGG--AAAGGAGGGGCAGGAGCCA--CC
human       AAAAACACGGG------AAGACGTGCGCGTGCGCG----------GCCGCCGCTGGGAGCCACC----AGGCGGCGGAGA-GGACAGCGACAGG---AAGGGAGGGGCCCGAGCCA--CC
macaque     AAAAACACGGG------AAGACGTGCGCGTGCGCG----------GCCGCCGCTGGGAGCCACC----AGGCGGCGGAGA-GGACAGTGGCAGG---AAGGGAGGGGCCCGAGCCA--CC
marmoset    AAAAACACGGG--AAGACGGACGTGCGCGTGCGCG----------GCCGCCGCTGGGAGCCACC----AAGCGGCAGAGA-GGACAGCGGCAAG---GAGGGAGGGGCCCAATCCA--CC
bushbaby    AAAGACACAGG------AGAACGTGCGCGTGCGCG----------GCGGCCGCTCGGAGCTATC----AGGCGGCTGAGA-GGACAG----AGG---GAGGGAGGGGCTGGAGCCT--TC
pig         AAAGACACGGG------AGGGCGTGCGCGTGCACGCGCG------GCCGCCGCTCGGAGTTACC----AAGCCGCAGAGA-GGACGG----AGA---AAGGGAGGGGCAGGAGCTA--CC
dolphin     AAAGACTCGGG------AGGGCGTGCGCGTACGCGTGCGCGCCCGGCCGCGGTTCTGAGCTACC----GAGCGGCTGAGA-GGAC------AGA---GAGGGAGGGGCAGGAGCTA--CC
cow         AAAGAGTCGGG-------AGGGCTGCGCTTGCGCTCCCG------GCCGCCGCTCTGAGCTACC----GAACAGCCAAGA-GGACAG----AGA---GAGGGAGGGGCAGGAGCCA--CC
horse       AAAGACACGGG------AGGGCGTGCGCGTG--------------GCCGCAGCTCTGAGCTACC----AGGCGACTGAGA-GGACAG----AGA---GAGGGAGGGGCAAGCGCCA--CC
megabat     AAAGACACGGG------CGGGTGTGCGCGCAC-------------GGCGCCGCTCTGAGCTATATCAGAGGCGGATGAGA-GGACAG----ACAC--GGGGGAGGAGCAGGATCCG--CG
cat         AAAGACATGGG------TGTGCGTGCGCGTGCGCG----------GTCACCGCTCGGAGCTACC----AGGAGGCTGAGA-GGACAC----CGA---GAGGGCGGGGCAGGAGCTA--CC
dog         AAAGCCATGGGCAGGCGTGCGCGTGCGCGTGCGCG----------GCCACCGCTCGGAGCTACC----AGGAGGCTGAGG-GGACGC----AGA---GCGGGAGGGGCAGGAGCCG--CC
ferret      AAAGACATGGG------CAGGCGTGCGCGTGCGCG----------GCCACAGCTCGGAGCTACC----AGGAGGCTGAGA-GGACAC----AGA---GCGGGAGGGGCAGGAACCA--CC
microbat    AAAGAGAGGGG------CGGGCGTGCACGCGCG------------GCCGCGGCTCTGAGCTCCT--AGAGGCGGCCGAGA-GGACAG----AGA---GAGGGAGGGGCAGAAGCCA--CC
tenrec      AAAGCCTCGGA-----AGAAGCGTGCGCGTGCGTG----------GTCGTCGCGCTGCGCTACC----AGGCGGCGGAGA-GTACAG-----CGA--GAGGGAGGGGCTGGTGTCAGACC
guinea pig  CAGGACGCGGG------AGAGCGTGCGCGTGCGCG----------GCCGCCGCTCTACGTTACC----GGGCATTAGAAAAGGACAGAGG-GAGC--GAGGGAGGGGCTGGAGCCG---T
hedgehog    AAAAAGACTGG------AGGCCGTACGCGTGCGCGCGCGAGA--GACCGCCGCTCCGAGCTGCCC----GGCAGCCGAGG-GGACGC----GGGTCGGAGGGAGGGGCAGGAGTCGAGCC
            ^^^      *           ^^*^^^*^^^^             ^ ^   *^^  ^^*   ^^      ^     ^*^  *^**               ** **^*^  ^       ^^

                                                                                            Sp1          Sp1
rabbit      GACCG---------AAGGAGGAGGAGGAGGAGGAGAAAGGGTTGTGCTCTTGGCC----------------GAGCGAGGGGCGGGGCCG-CGGGCGGCGCGTG--CAGGTGCCGAGCTC
pika        GACCGC------AGGAGGAGGAGGAGGAGGAGGAGAAAGGGTTGTGCTCTCGGCC----------------GAGCGAGGGGCGGGGCCG-CGGGCGGCGCGAG--CAGGTGCAGGGCTA
mouse       GGCCGCGGCGGGAGGAGGAGGAGGAGGAGGAGGAGACGGGGTTGTGCTCCTGGGCAG---------CTGAGGAGCGAGGGGCGGGGCCGGCGAGCGGCGCGTG--CAGGTGCCAGGCTC
human       GACTGC------------------AGGAGGA-GAAG--GGGTTGTGCTCCTGGCCGA---------GGAAGGAGAAAGGGGCGGGGCCGGCGGGCAGCGTGCG--GCAGTGCGGAGCTC
macaque     GACTGC------------------AGGAGGA-GAAG--GGGTTGTGCTCCTGGCCGA---------GGAAGGAGAAAGGGGCGGGGCCGGCGGGCAGCGCGCG--GCGGTGCGGAGCCC
marmoset    GACTGC------------------AGGAGGAAGAAG--GGGTTGTGCTCCTGGCCGA---------GGAAGGAGAAAGGGGCGGGGCCGGCGGGCAGCGCGCG--GCGGTGCGGAGCGC
bushbaby    GACAGC------------------TGGAGAAGGAGACCGGGTTGTGCTCCTGAGCGC---------GGAAGGAGAAAGGGGCGGGGCCGGCGGACAGTGTGTG--AAGGTGCCGAGCTC
pig         GACCAA------------------AGGAGGAGGAGAAGGGGTTGTGCTCCTGACCTA---------GCGAGGAGAAAGGGGCGGGGCCGTCCAGCTGCGCGCG--GAGGTGCAGAGCTC
dolphin     GACCGC------------------AGGAGGAGGAGAAGGGGTTGTGCTCCTGGCCGA---------GCAAGGAGAAAGGGGCGGGGCTGGAGAGCAGCGCGCC--GGGGTGCCGAGCTC
cow         GACCGC------------------CGGAGGA---GAAGGGGTTGTGCTCCTGGACGA---------GCGAGGAGAAAGGGGCGGGGCCGGCGAGCAGCGCGCG--GAGGTGCACCGCTC
horse       GACCGC------------------AGGAGGAGGAGAAGGGGTTGTGCTCCTGGCCGA---------GCGAGGAGAAAGAGGCGGGGCCGGCAAGCAGTGCGTG--GAGGTGCCGAGCTC
megabat     GACCGC------------------TGGAGGAGGAGAAGGGGTTGTGCTCCCGGCCGT---------GCGAGGAGAAGGGGGCGGG-CCGGCGACCGGCGCGTG--AAGAGGCCGAGCTC
cat         GACCGC------------------TGGAGGAGGA-AAGGGGTTGTGCTCTTGGCCGA---------GCGCGGAGAAAGGGGCGGAGCCTGCGAGCAGCGCGTG--GAGGTGCCGAGCTC
dog         GACCGC------------------AGGAGGAGGA-AAGGGGTTGTGCTCCTGGCCGA---------GCGAGGAGAAAGGGGCGGGGCCGGCGGGCAGCGCGTG--GAGGTGCCGAGCTC
ferret      GACCGC------------------TGGAGGAGGA-AAGGGGTTGTGCTCCTGGCCGA---------GCGAAGAGAAAGGGGCGGGGCCGGCGAGCAGCGCGTG--GAGGTGCCGAGCTC
microbat    GACCTC------------------AGGAAG-GGAGAAGGGGTTGTGCTCTTGGCCGC---------ACGCAGAGAAAGGGGCGGGGCCGGCGGGCGGGGCGCG--AAGGTGCCGAGCTC
tenrec      GGCCGC------------------TGGAGAAG------AGGTTGTGCTCTTCGCCTC---------GGAAGGAAGAAGGGGCGGGGCCGGCGGGCCGCGCGTGTGGAGGTGCCGGGCTC
guinea pig  GACC-C----------------------GGCGGAGAAGGCGCTGTGCTCCC-GCCGA---------CGGAAGGGCAGGGGGCGGGGCCGGCGGGCGGCGCGTG--CAGGTGCCCTGCGG
hedgehog    GGCGGCC---------------GCAGGAGGCGGAGA-GGGGTTGCGCTCTCAGCCGGCCACAGGCGGCAACGAGGAAGGGGCGGGGCCG--------CGCGCC--GAGGGGCCGAGC-C
            *^*  ^                   ^^^ ^  ^^    ^^*^**^****   * *                *^^  ^*^*****^^*^^ ^  ^^ ^ *^* ^    ^^^**   ** ^
```

```
                     Sp1                            Sp1
rabbit      CTGGGACCCG-C-CGGCGTGCGGG-GGGTCT-GTGG-CCCTCGCCGTCGAAGCA---GTCGCCGCCGTCGCTG------GGTCG
pika        CCGGGACCCG-C-CGGCGCGCGAG-GG-TCC-TTGG-CCCTCGCCGTCGCAGCAA--GTGGCCGCCGTCGCTG------GGTCG
mouse       CCGGGCTCCGGC-TGGCGCGCGAGCGGCTCC-GTGGGCCCCCGCCGCCGCAGTGGCAGTGGCCGCCGCCGCCGCTTGGTCG
human       CTGAGA-CCGGC-GGGCACACGGG--GGTCT-GTGG-CCCCCGCCGTAGCAG------TGGCTGCCGCCGTCG---CTTGGTT-
macaque     CTGAGA-CCGGC-GGGCACACGGG--GGTCT-GTGG-CCCTCGCCGTCGCAG------TGGCCGCCGCCGTCG---CTTGGTT-
marmoset    CGGAGG-CCGGC-GGACAGGCGGG--GGTCT-GTGG-CCCTAGCCGTCGCAG------TGGCCGCTGCTGTCG---CTTGGTTG
bushbaby    TCGCGG-CCGGC-CGGCGCGCGGG--CGTGT-GTGG-CCCTAGCCGTCGCAG------TGGCCGTCGCCGTCC---TTTGGTC-
pig         CTGGGA-CCGGC-GGGCGCGCGGG--GGTCTCGTGG-CCCTTGCCCTCCCAG------TAGCCGCCGCCGTCG---CTTGACCG
dolphin     CTGGGA-CCGGC-GGGCGCGTGGG--GGTCT-GTGG-CCCTTGCCGTCCCAG------TGGCCGCCGCCGTCG---CTTGGCTG
cow         CTGGGA-CCGG--GGCCGCCCGGG--GGTCT-GTGG-CCCTTGCCGTCCCAG------TGGCTGCCGCCGTCG---CTTGGCCG
horse       CTGGGA-CCGGC-GGGCGCGCGGG--GGTCT-GTGG-CCCTCGCCGTCCCAG------TGGCCGCCGCCGTCG---CTTGGTCG
megabat     CCGGGG-CCGGC-CGGCGCGCGGT--AGTCT-GTGG-CCCTCGCTGTCCCAG------TATCCGCTGCCGTCG---CTTGGTCG
cat         CTGGGA-CCGGC-GGGCGCGCGGG--CGTCT-GTGG-CCCTCGCCGTCCCAG------TGGCCGCCGCCGTCG---CTTGGTCG
dog         CTGGGC-CCGGC-GGGCGCGCGGG--CGTCT-GTGG-CCCTCGCCGTCCCAG------TGGCCGCCGCCGTC----CTTGGTCG
ferret      TCGGAA-C-GGC-GGACGCGCGGG--CGTCT-GCGG-CCCTCGCTGTCCCAG------TGGCCGCCGCCGTGG---CTTGGTCG
microbat    CCGGGA-CCGGC-CGGCGCGCGGG--GCTCT-GCGG-CCCTCGCCGTCCCAG------TGGCCGCTGCCGTCA---TTTGGTCG
tenrec      TAGGGA-CCGGC-GGGCTCGCGGG--CGTCC-GTGG-CCGTCGCT-TC-----------GGCTGCTGCCGTCG---CCTGGTCG
guinea pig  CTGAGC----------CCCGCGGG--GGTCC-GTGG-CCCTCGC-GTGGCAG------TGGCCGCCGCTGAAG---CTTGGTCG
hedgehog    CCGGGA-CCGGCGGGGGGCGCGGG-------------CCCTCGTCGTGCCGG-------GGCCACCACCGCCG---CTCGGTGG
              * ^  ^^^ ^  ^ ^ ^ ^*^^    ^^  ^ ^^ **^^ *^ ^^  ^^^      ^ ^* ^^ ^^^*         *^

                                   Sp1                                     INIT
rabbit      ----CCGTCGGTCTGCGGA-AGGCGGGTTATGGCGGCGGCGGC---AGTGGGAGCTGTGAATGAATTCTCCGGGTGGACGA
pika        ----CCTTGGGTCTGCGGG-AGGCGGGTTATGGCGGCGGCGGCGGCAGTGGAAGCCGTGAATGAATTCTCCGGGTGGACGA
mouse       ----CCGTCGGTCTGCGGG-AAGCGGGTTATGGCGGCGGCGGC------AGTGGGAGCTGTGAATGAGTTCTCCGGCCGGACGA
human       ---CCCGTCGGTCTGCGGG-AGGCGGGTTATGGCGGCGGCGGC------AGTGAGAGCTGTGAATGAATTCTCCGGGTGGACGA
macaque     ---CCCATCGGTCTGCGGG-AGGCGGGTTATGGCGGCGGCGGC------AGTGAGAGCTGTGAATGAATTCTCCGGGTGGACGA
marmoset    GTTCCCGTTGGTCTGCGGG-AGGCGGGTTATGGCGGCGGCGGC------AGTGAGAGCTGTGAATGAATTCTCCGGGTGGACGA
bushbaby    ---CCCGTCGGTCTGCGGG-AGGCGGGTTATGGCGGCGGCGGC------AGTGGGAGTTGTGAATGAATTCTCCGGGTGGACGA
pig         ----CCGTCGGTCTGCGGA-AGGCGGGTTATGGCGGCGGCGGC------AGTGGGAGCTGTGAATGAATTCTCCGGGTGGACGA
dolphin     ----CCGTCGGTCTGCGGG-AGGCGGGTTATGGCGGCGGCGGC------AGTGGGAGCTGTGAATGAGTTCTCCGGGTGGACGA
cow         ----CCGTCGGTCTGCGGG-AGGCGGGTTATGGCGGCGGCGGC------AGTGGGAGCTGTGAATGAATTCTCCGGGTGGACGA
horse       ----CCGTCGGTCTGCGGGGAGGCGGGTTATGGCGGCGGCGGC------AGTGGGAGCTGTGAATGAATTCTCCGGGAGGACGA
megabat     ----CCGTCGGTCTGCGGA-AGGCGGGTTATGGCGGCGGCGGC------AGTGGGAGCTGTGAATGAATTCTCCGGGTGGACGA
cat         ----CCGTCG-TCTGCGGG-AGGCGGGTTATGGCGGCGGCGGC------AGTGGGAGCTGTGAATGAATTCTCCGGGTGGACGA
dog         ----CCGTCGGTCTGCGGG-AGGCGGGTTATGGCGGCGGCGGC------AGTGGGAGCTGTGAATGAGTTCTCCGGGTGGACGA
ferret      ----CCGTCGGTCTGCGGG-AGGCGGGTTATGGCGGCGGCGGC------AGTGGGAGCTGTGAATGAATTCTCCGGGTGGACGA
microbat    ----CCGTCGGTGTGCGGG-AGGCGGGTTATGGCGGCGGCGGC------AGTGGGAGCTGTGGATGAATTCTCCGGGTGGACGA
tenrec      ----CCGTCGGTCTGCAGG-AGGCGGGTTATGGCGGCGGCGGC------AGTGGGGGCTGTGAATGAATTCTCCGGGCGGACGA
guinea pig  ----CCGTCGGTCTGAGGG-AGGCAGGTTATGGCGACGGCGGC------AGTGGGAGCTGTGAATGAGTTCTCCGGGTGGACGA
hedgehog    ----CCCTGGCTCT---------CGGGTTATGGCGGCGGCGGC------AGCGGGGGCTGTGAATGAATTCTCCCGGCCGACGA
                ** * *^*^*^^^^  ^^^*^**********^*******      **^* ^^*^^***^**** ******^*^ ^*****
```

B

```
                                            NRF1         NRF1                        Sp1
tenrec     GGAACTGCAGTTCCGCGGGGAA-TGG-GCCGTGCAGCAGGCACT-GCACAC---------------GCGCATTAGA-------------------GGA
elephant   TGACCGGCAGTTACACTGGGAACTGGCGCCGCTCA-CGCGCACTAGAACCCAGGTCGACGAAGATGGCGCTTTAGAAAGCCGCCCAAGAAATACATGGA
            ** * ****** * * ***** *** ****   ** *   ***** * ** *                **** *****                 ***

                 NRF1         SOX11                         NRF1                               NRF1
tenrec     A------GCCCG-----GCCAAC-AAAGAC------GGCGGCGTGTGCGCGTGCGTGCGCCGGAGAAAGCCTCGGAAGAAG---CGTGCGCGTGCGTG-
elephant   AAGACGTGCGCGTGCGCGCCAGTGAAAGACACGAGGGGAGGGGCGTGCGCGTGCG--CGCCAGGGAGAGACACGGAGGAAGAGACGTGCGCGTGCGCGC
           *       ** **      ****    ******      ** ** * ***********  **** * ** ** * **** ****   *********** *

tenrec     -GTCGTCGCGCTGCGCTACCAGGCGGCGGAGAGTACAGCGAGAGGGAGGGG-----------CTGGTGTCAGACCGGCCGCTGGAGAAGAGGTTGTGCT
elephant   AGCTGTAGCACTGAGCCACCAGGCACTCCGGAAGACAGAGGGAGGTAGGGGTGGAGCAACTGCTGGTGTCAGACTC-CCGCATGAAAATGGCTTGTTCT
            *  ** ** *** ** *******     **  **** * **** *****             ************   ****  ** **  * **** **

                                     Sp1     Sp1                                Sp1
tenrec     CTTCGCCTCGGAAGGAAGAAGGGGCGGGGCCGGCGGGCCGCGCGTGTGGAGGTGCCGGGCTCTAGGGACCGGCGGGCTCGCGGGCGTCCGTGGCCGTCG
elephant   TCT-------GACTGAAGAAG--------CCGGCGGGCAACGCGT--GGAGCTGCCGAGCTCTGGGG-CCAGCGGGCGCGCGGGGGTTTGTGGCCATCG
             *       **  *******         *********  *****  **** ***** ***** *** ** ****** ****** **  ****** ***

                                                       Sp1                            INIT
tenrec     CTTCGGCTGCTGCCGTCGCCTGGTCGCCGTCGGTCTGCAGGAGGCGGGTTATGGCGGCGGCGGCAGTGGGGGCTGTGAATGAATTCTCCGGGCGGACGA
elephant   CCGTGGCCGCCGCCGTGGCTTGGTCATCGTGGTTCTGCGGGAGGCGGGTTATGGCGGCGGCGGCAGTAGGAGCTGTGGATGAATTCTCCGGGTGGACGA
           *   *** ** ***** ** *****  *** * ***** **************************** ** ****** ************* ******
```

C

```
                 NRF1                        SOX11                            NRF1                  
hedgehog    GCAGCACATGCGCACTA--GGTGTTCAGGGCAACAAAGACTAAGGCGTGC---------------GCT----------------TGCGCAT-------
Monodelphis GCTGCACAAGCGCACCGCGGGAGCTCCTGTCAACAAAGACCGAGGCACGCTCGTGAGCAACGGAGGCTAAGATCCTATGGAACGTGCGCGTGTCCGCC
            ** ***** ******    ** * **   * **********  ****  **               ***                ***** *

                                                  NRF1
hedgehog    ----TGAGG------ATGAAAGAAAAAAAAAAAAGACTGGAGGCCGTACGCGTGCGCGCGCGAGAGACCGCCGCTCCGAGC---TGCCCGGCAGCCGA
Monodelphis TCGCTGAGGCAGCCTATGAAAGGAGAAAGAAAG-GACAGGA---------CGTGCGCGCGACTGTC-CCATTGC-CTGAGCCTATGGCCGTGAGA-GA
                *****       ******* * *** ***  *** ***          **********   *   **    ** * ****   ** ***  **  **

                  Sp1
hedgehog    GGGGACGCGGGTCGGAGGGAGGGGCAGGAGTCGAGC--CGGCGGCCGC--AGGAGG----CGGAGAGGGGTTGCGCTCTCAGCCGGCCACAGGCGGCAA
Monodelphis GGAGG-GCGGGGCGGGACCAGGA-CAGATCCCGGACATCAAAACCTGCTCGGGAGGGTTTCGGAGAGGGG--GAGGTGAC-----GCTTCGGACCG---
            ** *  ***** ***    ***  ***    **   *   *      * **    *****    **********  * * *  *      **  * * * *

                 Sp1
hedgehog    CGAGGAAGGGGCGGGGCCGCGCGCCGAGG-GGCCG-AGCCCCGGGA------CCGGCGG------------GGGGCGCGGGCC--------CTCGTCGT
Monodelphis -AGGGAAGGGGCGGGGCCGGGTGTGGAGGTAGCTGGAGCTCTGAGGACTCGCCCGACTGCTACCCAGCTTTGGTGCTTTGCCCTTCTCCTCCTCCTCTT
               *************** * *  ****  ** * *** * * *         *** * *               ** **    * **         *** ** *

                                                                                       INIT
hedgehog    GCCGG-GGC-----CACCACCGCCGCTCGGTGGCCCTGGCTCTCGGGTTATGGCGGCGGCGGCAGCGGGGGCTGTGAATGAATTCTCCCGGCCGACGA
Monodelphis GCCTGCAGCACCAGCACCACCGCCGCTTGGTCG--CTGACAG-CGGGTTATGGCGGCGGCGGCAGTGGGAGCCGTGAATGAATTCTCCGGGCGGCCGA
            *** *  **      ************ *** *   *** *   ********************* *** ** ************** *** * ***
```
