# Supplementary material for: Transcriptional and Post-Transcriptional Regulation of SPAST, the Gene Most Frequently Mutated in Hereditary Spastic Paraplegia
Source: PLoS One. 2012 May 4;7(5):e36505. doi: 10.1371/journal.pone.0036505 (PMC3344893; doi:10.1371/journal.pone.0036505)

Figure S2. An extended region upstream of the *SPAST* promoter that includes a putative Elk1 binding site, has minor effects on transcriptional activity. A fragment containing the putative Elk1 site (Canbaz et al. 2011) was ligated into the pGL3b vector to generate pGL3b-Elk1 and into the pGL3b-SPAST-promoter construct (the "full-length promoter" construct in **Fig. 5A**) to generate pGL3b-SPAST-promoter-Elk1, which are depicted in the panel on the left. Luciferase assays were performed and the data was normalized to the pGL3b vector, as presented in the panel on the right. Inclusion of the fragment with the Elk1 site led to a significant increase (9-fold) in luciferase activity in pGL3b-Elk1 as compared to pGL3b, but this is significantly less than that from the "full-length promoter" construct with 71-fold increased activity over pGL3b. Inclusion of the fragment with the Elk1 site into the pGL3b-SPAST-promoter construct led to a slight decrease in luciferase activity that was not significantly different than luciferase activity in cells transfected with pGL3b-SPAST-promoter.

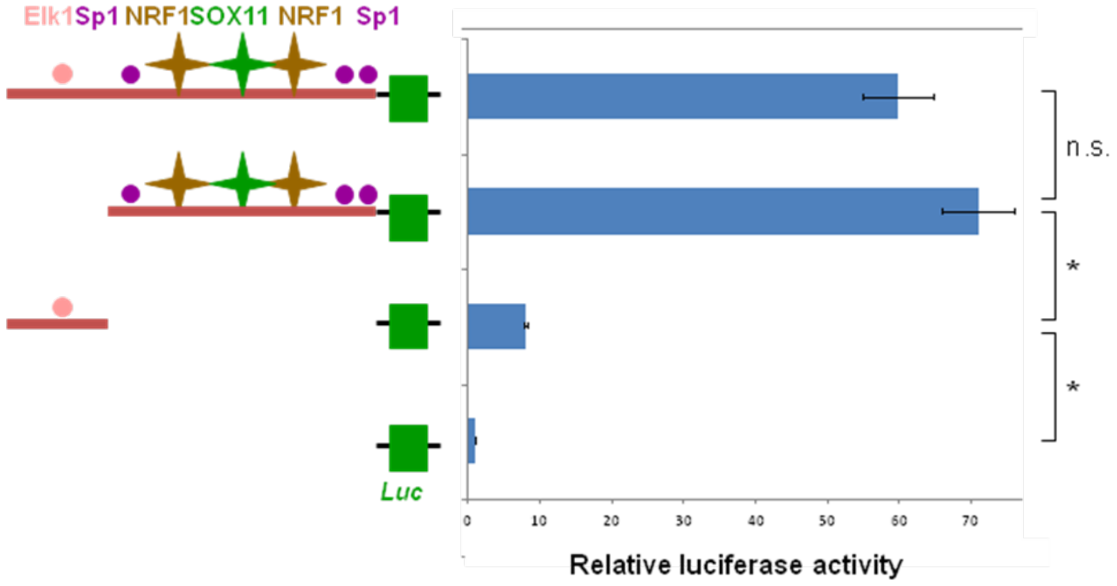

Supplement: Figure S2 — An extended region upstream of the SPAST promoter that includes a putative Elk1 binding site, has minor effects on transcriptional activity. A fragment containing the putative Elk1 site (Canbaz et al. 2011) was ligated into the pGL3b vector to generate pGL3b-Elk1 and into the pGL3b-SPAST-promoter construct (the “full-length promoter” construct in Fig. 5A ) to generate pGL3b-SPAST-promoter-Elk1, which are depicted in the panel on the left. Luciferase assays were performed and the data was normalized to the pGL3b vector, as presented in the panel on the right. Inclusion of the fragment with the Elk1 site led to a significant increase (9-fold) in luciferase activity in pGL3b-Elk1 as compared to pGL3b, but this is significantly less than that from the “full-length promoter” construct with 71-fold increased activity over pGL3b. Inclusion of the fragment with the Elk1 site into the pGL3b-SPAST-promoter construct led to a slight decrease in luciferase activity that was not significantly different than luciferase activity in cells transfected with pGL3b-SPAST-promoter. (PDF) [file pone.0036505.s003.pdf]
