# Supplementary material for: Transcriptional and Post-Transcriptional Regulation of SPAST, the Gene Most Frequently Mutated in Hereditary Spastic Paraplegia
Source: PLoS One. 2012 May 4;7(5):e36505. doi: 10.1371/journal.pone.0036505 (PMC3344893; doi:10.1371/journal.pone.0036505)
Supplement: Figure S3 — Evolutionary establishment and maintenance of miR-367 targeting of SPAST mRNA in primates. A) Base-pairing of the Homo sapiens (hsa) miR-367 miRNA with the SPAST 3′-UTR. The optimal seed motif (green text and yellow shade) for targeting by the miRNA is at position 1,594–1,600 of the SPAST 3′-UTR for human, and similarly the optimal seed motif is maintained in chimpanzee, orangutan, and gibbon (see Fig. S3C). In macaque and baboon, the SPAST target:miR-367 seed pairing includes a U:G pair (see Fig. S3C). Additional sequences that can contribute to the SPAST target:miR-367 pairing outside the seed are shown by gray shade. B) The miRNA seed motif for miR-367 is shared with six other miRNAs, any of which could target the SPAST 3′-UTR in vivo. At top, the human SPAST 3′-UTR sequence that is targeted by miR-367 is displayed, followed underneath by the 7 mature miRNA sequences (from miRBase) that would be capable of targeting the SPAST mRNA (yellow shade, miRNA seed and target sequences; gray shade, nucleotides that can contribute to auxiliary pairing, without gaps). C) Multisequence alignment in primate species for the boundaries of the Alu element insertion in the SPAST 3′-UTR, highlighting the 18-nt target site duplication (TSD; bold blue and underline) and the miR-367 target site (yellow shade, target sequence for optimal miR-367 seed; gray shade, auxiliary pairing). D) Multisequence alignment in mammals for the position of the Alu element insertion in the SPAST 3′-UTR, highlighting the 18-nt TSD present in catarrhine primates and single copy in the other mammalian species. In C) and D): *, conserved in all species;∧, conserved in 13/14 species;+, presence of the Alu element insertion; - (in species name), absence of the Alu element insertion; ##Alu## represents the remaining Alu element sequences, not shown for illustrative purposes; Tn represents the number of T nucleotides at that position. E) Primate phylogenetic tree showing the presence (+) or absence (-) of an A [file pone.0036505.s004.pdf]

Figure S3. Evolutionary establishment and maintenance of miR-367 targeting of *SPAST* mRNA in primates. A) Base-pairing of the *Homo sapiens* (hsa) miR-367 miRNA with the *SPAST* 3'-UTR. The optimal seed motif (green text and yellow shade) for targeting by the miRNA is at position 1,594-1,600 of the *SPAST* 3'-UTR for human, and similarly the optimal seed motif is maintained in chimpanzee, orangutan, and gibbon (see **Fig. S3C**). In macaque and baboon, the *SPAST* target:miR-367 seed pairing includes a U:G pair (see **Fig. S3C**). Additional sequences that can contribute to the *SPAST* target:miR-367 pairing outside the seed are shown by gray shade. **B)** The miRNA seed motif for miR-367 is shared with six other miRNAs, any of which could target the *SPAST* 3'-UTR *in vivo*. At top, the human *SPAST* 3'-UTR sequence that is targeted by miR-367 is displayed, followed underneath by the 7 mature miRNA sequences (from miRBase) that would be capable of targeting the *SPAST* mRNA (yellow shade, miRNA seed and target sequences; gray shade, nucleotides that can contribute to auxiliary pairing, without gaps). **C)** Multisequence alignment in primate species for the boundaries of the Alu element insertion in the *SPAST* 3'-UTR, highlighting the 18-nt target site duplication (TSD; bold blue and underline) and the miR-367 target site (yellow shade, target sequence for optimal miR-367 seed; gray shade, auxiliary pairing). **D)** Multisequence alignment in mammals for the position of the Alu element insertion in the *SPAST* 3'-UTR, highlighting the 18-nt TSD present in catarrhine primates and single copy in the other mammalian species. In **C)** and **D)**: *, conserved in all species; ^, conserved in 13/14 species; +, presence of the Alu element insertion; - (in species name), absence of the Alu element insertion; ##Alu## represents the remaining Alu element sequences, not shown for illustrative purposes; T_n represents the number of T nucleotides at that position. **E)**

Primate phylogenetic tree showing the presence (+) or absence (-) of an Alu repetitive element insertion in the *SPASTIN* 3’-UTR, that includes a miR-367 optimal seed motif for targeting by the miRNA. An * indicates species in which the pairing of miR-367 with its target includes a G:U pair. The putative origin of the Alu element is shown by a red arrow.

A

```
  human SPAST 3′-UTR     5′...GCUCUUGUUGCCCAGGCUGGAGUGCAAUGGC...
                                   |::||    |||::||||||||
            hsa-miR-367          3′-AGUGGUAACGAUUUCACGUUAA-5′

macaque SPAST 3′-UTR     5′...GCUCUUGUUGCCCAGGCUGGAGUGUAAUGGC...
                                   |::||    |||::||||:|||
            mml-miR-367          3′-AGUGGUAACGAUUUCACGUUAA-5′
```

B

Human *SPAST* 3’-UTR   5′-UCUUGUUGCCCAGGCUGGA**GUGCAAU**GGCGC-3′

```
hsa-miR-367-3p:      44-aauugcacuuuagcaaugguga-65
hsa-miR-25-3p:       52-cauugcacuugucucggucuga-73
hsa-miR-32-5p:        6-uauugcacauuacuaaguugca-27
hsa-miR-92a-1-3p:    48-uauugcacuugucccggccugu-69
hsa-miR-92a-2-3p:    48-uauugcacuugucccggccugu-69
hsa-miR-92b-3p:      61-uauugcacucgucccggccucc-82
hsa-miR-363-3p:      50-aauugcacgguauccaucugua-71
```

C

```
human+       GAATATAGAAGATGCATGATTTCTGGGT(T14)GAGACAGAGTTTCGCTCTTGTTGCCCAGGCTGGAGTGCAATGGCGC##Alu##CCAGCCAGAAGATGCATGATTTCTTAGGAT
chimpanzee+  GAATATAGAAGATGCATGATTTCTGTTT(T17)GAGACAGAGTTTCGCTCTTGTTGCCCAGGCTGGAGTGCAATGGCGC##Alu##CCAGCCAGAAGATGCATGATTTCTTAGGAT
orangutan+   GACTATAGAAGATACATGATTTCTGGGT(T20)GAGACAGAGTTTCGCTCTTGTTGCCCAGGCTGGAGTGCAATGGCGC##Alu##CCAGCCAGAAGATGCATGATTTCTTAGGAT
gibbon+      GAATATGGAAGATGCATGATTTCTGGGT(T15)GAGACAGAGTTTCGCTGTTGTTGCCCAGGCTGGAGTGCAATGGCGC##Alu##CCGGCCAGAAGATGCATGATTTCTTAGGAT
macaque+     GAATATAGAAGATGCATGAAT-CTGG(T5CT6)GAGACGGAGTTTTGCTCTTGTTGCCCAGGCTGGAGTGTAATGGCGC##Alu##CCGGCCAGAAGAGGCATGATTTTTTAGGAT
baboon+      GAATATAGAAGATGCATGAAT-CTGG(T5CT6)GAGACGGAGTTTTGCTCTTGTTGCCCAGACTGGAGTGTAATGGCAC##Alu##CCGGCCAGAAGAGGCATGATTTTTTAGGAT
             ** *** ****** ***** * ***        ***** ****** *** *********** ******** ****** *       ** ********* ********* *******
```

D

```
human+       TTAAAGATTTG---TTTGAATATAGAAGATGCATGATTTCT##Alu##AGAAGATGCATGATTTCTT-----AGGATCATATGCTGTTTGTAGCCATAAGGTAAATCATGTCT
chimpanzee+  TTAAAGATTTG---TTTGAATATAGAAGATGCATGATTTCT##Alu##AGAAGATGCATGATTTCTT-----AGGATCATATGTTGTTTGTAGCCATAAGGTAAATCATGTCT
orangutan+   TTAAAGATTTG---TTTGACTATAGAAGATACATGATTTCT##Alu##AGAAGATGCATGATTTCTT-----AGGATCATATGCTGTTTGTAGCCATAAGGTAAATCATGTCT
gibbon+      TTAAAGATTTG---TTTGAATATGGAAGATGCATGATTTCT##Alu##AGAAGATGCATGATTTCTT-----AGGATCATATGCTGTTTGTAGCCATAAGGTAAATCATGTCT
macaque+     TTAAAGATTTG---TTTGAATATAGAAGATGCATGAAT-CT##Alu##AGAAGAGGCATGATTTTTT-----AGGATCATATGCTGCTCGTAGCCATAAAGTAAATCATGTCT
baboon+      TTAAAGATTTG---TTTGAATATAGAAGATGCATGAAT-CT##Alu##AGAAGAGGCATGATTTTTT-----AGGATCATATGCTGTTCGTAGCCATAAAGTAAATCATGTCT
marmoset-    TTAAAGATTTG---TCTGAATAT-------------------------AGAAGATGCATGATTTCTT-----AGGATCATATGCTGTTTGTAGCCATAAGTTAAATCATGTCT
pig-         TTAAAGATTTA---TCTCAACAC----------------------------AGATGTATGATCTCTT-----ACGGTCATATGCTATTTGTAGCCATAACTTAA-TCATGTCT
cow-         TTAAAGATTTA---TCTAAATGT-------------------------AGAAGATGTATGATT---------------ATATGCTGTTTGTAGCTATAACTTAAATCATGTCT
rat-         TTAAAGATTTAATACTTCAACAT-------------------------AGAAGATGTATTATTTCTT-----AGAATCATGCGCTGTTTTTAGCTGTAATTTAAATCACATCT
mouse-       TTAAAGATTTATTACTTAAATAT-------------------------AGAAGATGGATTATTTCTTTTCCTAGGATCATATGCTGTTTATAGCTGTAATTTAAATCATATCT
sloth-       TTAAAGATTTA----TCTAATTT-------------------------AGAAGATGTATGAATTCTT-----AGGATCAAGTGCTGTTTGTAGCCATTACTTAAATCACATCT
tarsier-     GTAAAGATTTA---TCTAAATAT-------------------------AGAAGATGTATGATTTCTT-----AAGATCATATGCTGTTTATAGTCATAATTTAAATTATGTCT
lemur-       TTTACTCTTGC---TCT---TGT-------------------------AGAAGACATATAATTTCTT-----AAGATCATACACTGTTTGTAGCCATAATTTGAATGATGTCT
             ^*^*^^^**^      ^ ^   ^                         ^^^*** ^ ** *^^^ ^^     ^   ^^*^    *  *  ***   * *  * * * *  ***
```

E

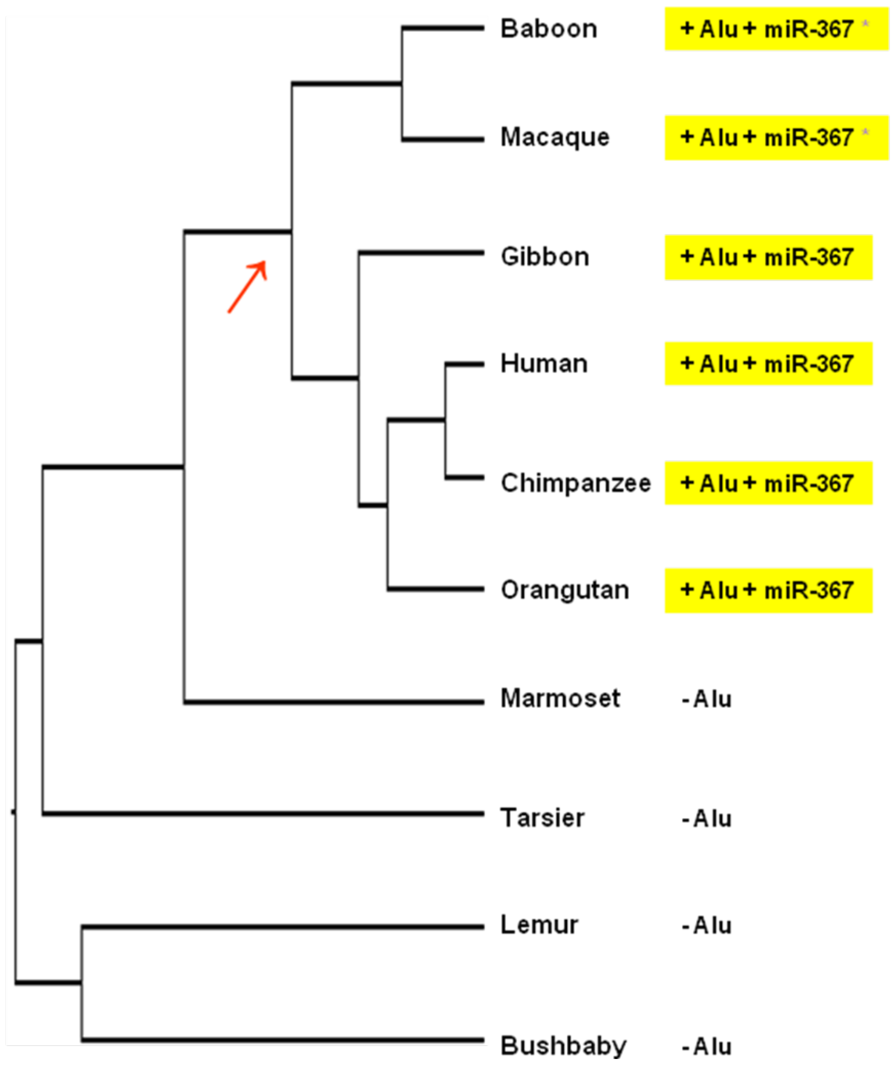
Baboon
+ Alu + miR-367
Macaque
+ Alu + miR-367
Gibbon
+ Alu + miR-367
Human
+ Alu + miR-367
Chimpanzee
+ Alu + miR-367
Orangutan
+ Alu + miR-367
Marmoset
- Alu
Tarsier
- Alu
Lemur
- Alu
Bushbaby
- Alu
