# Supplementary material for: Transcriptional and Post-Transcriptional Regulation of SPAST, the Gene Most Frequently Mutated in Hereditary Spastic Paraplegia
Source: PLoS One. 2012 May 4;7(5):e36505. doi: 10.1371/journal.pone.0036505 (PMC3344893; doi:10.1371/journal.pone.0036505)
Supplement: Table S1 — Conservation of SPAST 3′-UTR motifs as “optimal seeds” for miRNA targeting. (PDF) [file pone.0036505.s005.pdf]

Table S1. Conservation of *SPAST* 3'-UTR motifs as "optimal seeds" for miRNA targeting[a].

miRNA[b]	Optimal seed target	Pairing type	Frequency, eutherian[c]	Frequency, mars+mono[d]	Frequency, other tetrapods[e]
miR-96	GTGCCAA	8mer	100	100	100
miR-200bc/429	CAGTATT	7mer-m8	50	25	40
miR-132/212	GACTGTT	8mer	79	0	0
miR-30abcde/384	TGTTTAC	8mer	96	100	80
miR-29abc	TGGTGCT	7mer-m8	96	100	0

[a] Based on TargetScanHuman 5.2 (http://www.targetscan.org/), Ensembl (http://www.ensembl.org/index.html), and BLAST (http://blast.ncbi.nlm.nih.gov/Blast.cgi) analyses. Optimal miRNA pairing with an mRNA target utilizes nucleotides 2-8 of the mature miRNA.

[b] Each miRNA has a single optimal seed target (position 2-8 of the miRNA) in the human *SPAST* 3'-UTR, although for the miR-30abcde/384 seed several species have 2 or 3 optimal target sites in the *Spast* 3'-UTR. The conserved miR-30abcde/384 seed target in human *SPAST* 3'-UTR is not present in guinea pig, but another optimal target site is.

[c] Percentage of conserved targets among 28 eutherian mammal species, including human, horse, marmoset, rabbit, grey-mouse lemur, alpaca, cow, sheep, panda, guinea pig, bushbaby, sloth, shrew, ferret, rat, hamster, mouse, dog, American pika, tarsier, armadillo, elephant, microbat,western European hedgehog, kangaroo rat, squirrel, lesser hedgehog tenrec, and naked mole rat.

[d] Percentage of conserved targets among 3 marsupial (mars: South American opossum, tammar wallaby, and Tasmanian devil) and 1 monotreme (mono: platypus) species.

[e] Percentage of conserved targets among 5 other tetrapod species, including 3 birds (chicken, turkey, zebra finch), 1 lizard (green anole), and 1 amphibian (frog).
