# Supplementary material for: Transcriptional and Post-Transcriptional Regulation of SPAST, the Gene Most Frequently Mutated in Hereditary Spastic Paraplegia
Source: PLoS One. 2012 May 4;7(5):e36505. doi: 10.1371/journal.pone.0036505 (PMC3344893; doi:10.1371/journal.pone.0036505)
Supplement: Table S2 — PCR primers used in this study. (PDF) [file pone.0036505.s006.pdf]

Table S2. PCR primers used in this study.

Category	Primer #	Sequence
mouse *Spast* promoter F, ChIP	RN3345	5′-CAGAGAAAGGGGCATAGCAG-3'
mouse *Spast* promoter R, ChIP	RN3346	5′-CTCGGTGAATGGCTCTGGTG-3'
mouse *Snurf-Snrpn* promoter F, ChIP	RN2142	5′-GCAAAAATGTGCGCATGTG-3'
mouse *Snurf-Snrpn* promoter R, ChIP	RN2143	5′-CTCTCCTCTCTGCGCTAGTCTTG-3'
human *SPAST* promoter F, ChIP	RN3515	5′-TCTCCAGGGCTGCCGACGTGAG-3'
human *SPAST* promoter R, ChIP	RN3516	5′-TTCCTGTCGCTGTCCTCTCCGC-3'
human *SNURF-SNRPN* enhancer F, ChIP	RN3890	5′-GAAGGCTATGTCGAAATAACCTG-3'
human *SNURF-SNRPN* enhancer R, ChIP	RN3891	5′-CGGCTCTACAGGGAGGAAGC-3'
human *SPAST* F, qRT-PCR	RN3430	5′-GACTGATGGATACTCAGGAAG-3'
human *SPAST* R, qRT-PCR	RN3431	5′-GGCAGACATATTCTTCACCTG-3'
human *NRF1* F, qRT-PCR	RN2540	5′-GGAGTGATGTCCGCACAGAA-3'
human *NRF1* R, qRT-PCR	RN2541	5′-CCGTAGTGCCTGGGTCCAT-3'
human *SOX11* F, qRT-PCR	RN3896	5′-GTTCGACCTGAGCTTGAATTTC-3'
human *SOX11* R, qRT-PCR	RN3897	5′-tccttatccaccagcgacagg-3'
human *GAPDH* F, qRT-PCR	RN2547	5′-CGCCCCACTTGATTTTGG-3'
human *GAPDH* R, qRT-PCR	RN2546	5′-ATGGAAATCCCATCACCATCTT-3'
human *SPAST* promoter (*Mlu*I) F	RN3470	5′-GCTTACGCGTTGAGCCGAACTGCACATTGG-3'
Human *SPAST* promoter (*Xho*I) R	RN3471	5′-CATACTCGAGGTCTCAGGAGCTCCGCACTG-3'
Human *SPAST* 5'-promoter (*Kpn*I)[a] F	RN4113	5′-AAAAGGTACCGGCCAATTATCCTTCGGAG-3'
Human *SPAST* 5'-promoter (*Mlu*I) R	RN4114	5′-CATAACGCGTCGTCGGCAGCCCTGGAGAGC-3'

[a] PCR primer from Canbaz et al. (2011).

Canbaz D, Kırımtay K, Karaca E, Karabay A (2011) SPG4 gene promoter regulation via Elk1 transcription factor. *J Neurochem* 117: 724-734
